# Anti-TMV Effects of Amaryllidaceae Alkaloids Isolated from the Bulbs of *Lycoris radiata* and Lycoricidine Derivatives

**DOI:** 10.1007/s13659-018-0163-0

**Published:** 2018-05-18

**Authors:** Dong-Qiong Yang, Zhao-Rong Chen, Duo-Zhi Chen, Xiao-Jiang Hao, Shun-Lin Li

**Affiliations:** 1 State Key Laboratory of Phytochemistry and Plant Resources in West China, Kunming Institute of Botany, Chinese Academy of Sciences, Kunming, 650201 People’s Republic of China; 2 University of Chinese Academy of Sciences, Beijing, 100049 People’s Republic of China; 3 Tianjin Agricultural University, Tianjin, 300380 People’s Republic of China

**Keywords:** Amaryllidaceae alkaloids, Lycoricidine derivatives, *Lycoris radiata*, Tobacco mosaic virus, Anti-TMV activity

## Abstract

**Abstract:**

Fifteen known amaryllidaceae alkaloids were isolated from the bulbs of *Lycoris radiata*. Some of the compounds and lycoricidine derivatives had been screened for the activities against tobacco mosaic virus (TMV) by the conventional half-leaf method. Lycoricidine derivatives were also carried out the assay of effect on systemic infection of TMV by western-blot and RT-PCR analysis. The tested compounds showed moderate inactivation effect, whereas the lycoricidine derivatives showed good protective effect. The protective inhibitory activity of compounds L1 (*N*-methyl-2,3,4-trimethoxylycoricidine) (60.8%) and L3 (*N*-methyl-2-methoxy-3,4-acetonidelycoricidine) (62.0%) was almost similar to the positive control, Ningnanmycin (66.4%). RT-PCR and Western-blot analysis displayed that compounds L1, L3, L5 (*N*-allyl-2,3,4-triallyloxylycoricidine) exhibited antiviral activity, which was evidenced by reducing TMV-CP gene replication and TMV-CP protein expression. Additionally, defensive enzyme activities confirmed that compound L1 could increase the activity of PAL, POD, SOD to improve disease resistance of tobacco.

**Graphical Abstract:**

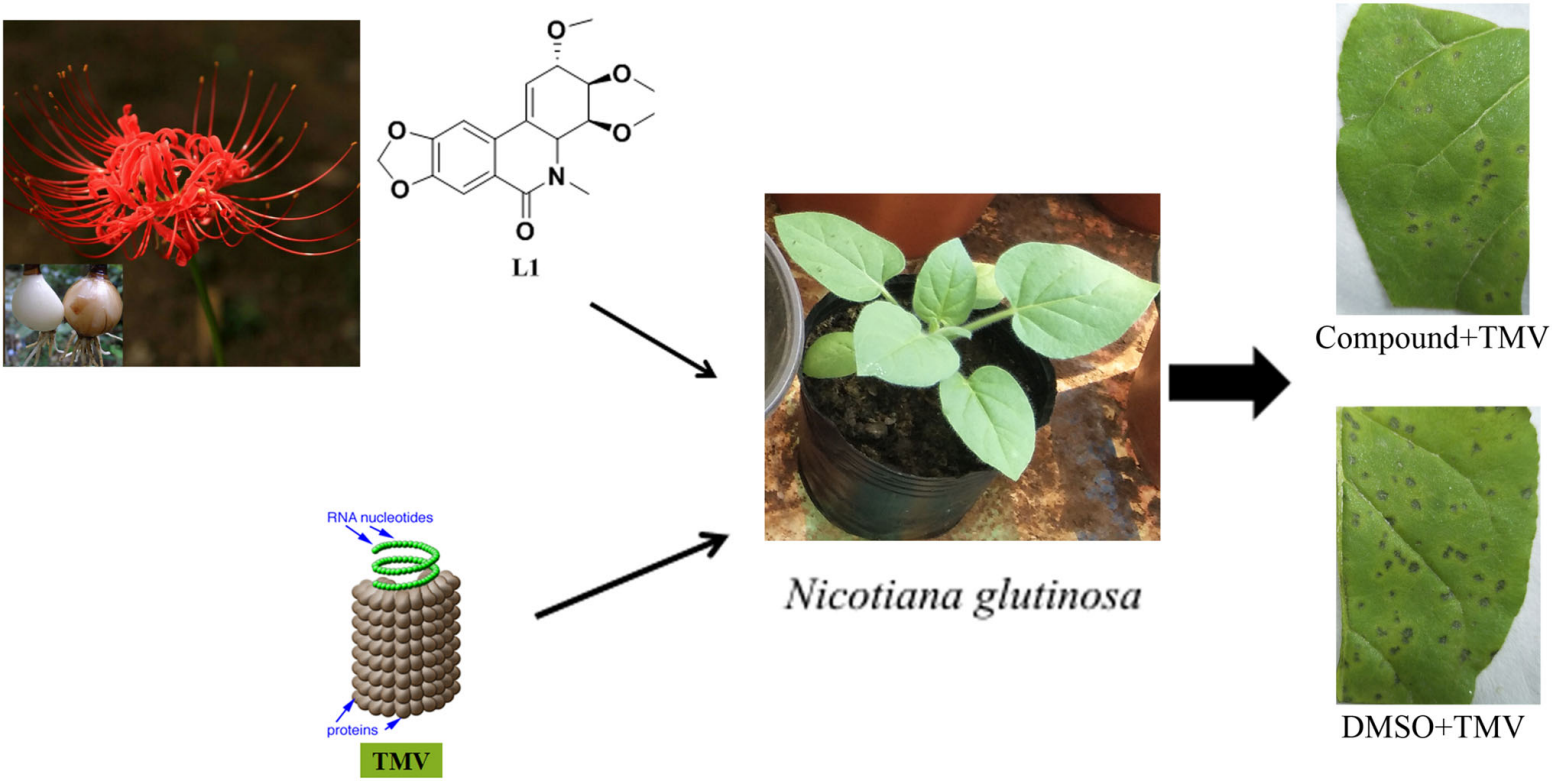

**Electronic supplementary material:**

The online version of this article (10.1007/s13659-018-0163-0) contains supplementary material, which is available to authorized users.

## Introduction

The viruses of plant cause significant loss in agriculture and horticulture and have the name “plant cancer” [1]. Tobacco mosaic virus (TMV) is regarded as a model virus and is widely studied, due to it contributes to plant pathology and economic importance. The symptoms of TMV infection include leaf distortion, height reduction, fruit deformation and low germination percent of seeds in tobacco. TMV is responsible for devastating diseases in major agricultural crops, including vegetables and tobacco, which leads to serious damage and enormous economic loss [2]. So far few effective anti-TMV agents are highly efficient. Therefore, low-priced agents that can control such diseases effectively are in great need.

In recent years, a series of natural products from plants and synthesised chemical compounds with anti-TMV activities had been reported, such as *seco*-pregnane steroids [3], 3-acetonyl-3-hydroxyoxindole [4], 7-deoxy-*trans*-dihydronarciclasine (DDN) [5], eudesmanolides [6], quassinoids [7], sesquiterpenoids [8], phenanthroindolizidines and their analogues [9], β-carbolines alkaloids [10] and their derivatives [11]. Among those compounds, DDN attracted our attention. DDN, a lycoricidine alkaloid, showed strong activity against TMV by the half-leaf method, the IC_50_ was 1.80 μM [5]. Our group had also found that lycoricidine derivatives exhibited potent inhibition of Hepatitis C Virus (HCV) [12]. Lycoricidine alkaloids were found in the plant of Amaryllidaceae and Liliaceae. *Lycoris radiata,* amaryllidaceae plant, is widely distributed in the South of China. It has been cultivated not only as ornamental plants for their colorful flowers, but also as folk herbal medicine against various diseases in many countries and areas, and their diverse activities have also been reported, such as anti-TMV [5, 13], antitumor, and acetylcholinesterase inhibitory activities *ect* [14].

To further assess whether lycoricidine derivatives have the same effects on TMV when compared to HCV. In our ongoing studies, we did some research about the chemical constituents of *Lycoris radiata* and the anti-TMV activities of amaryllidaceae alkaloids and lycoricidine derivatives, as well as defensive enzyme activities of the compound L1. The result suggested that lycoricidine alkaloids could enhance the resistance of plants against TMV and could be considered as a potential antiviral agent.

## Results and Discussion

**Fig. 1:**
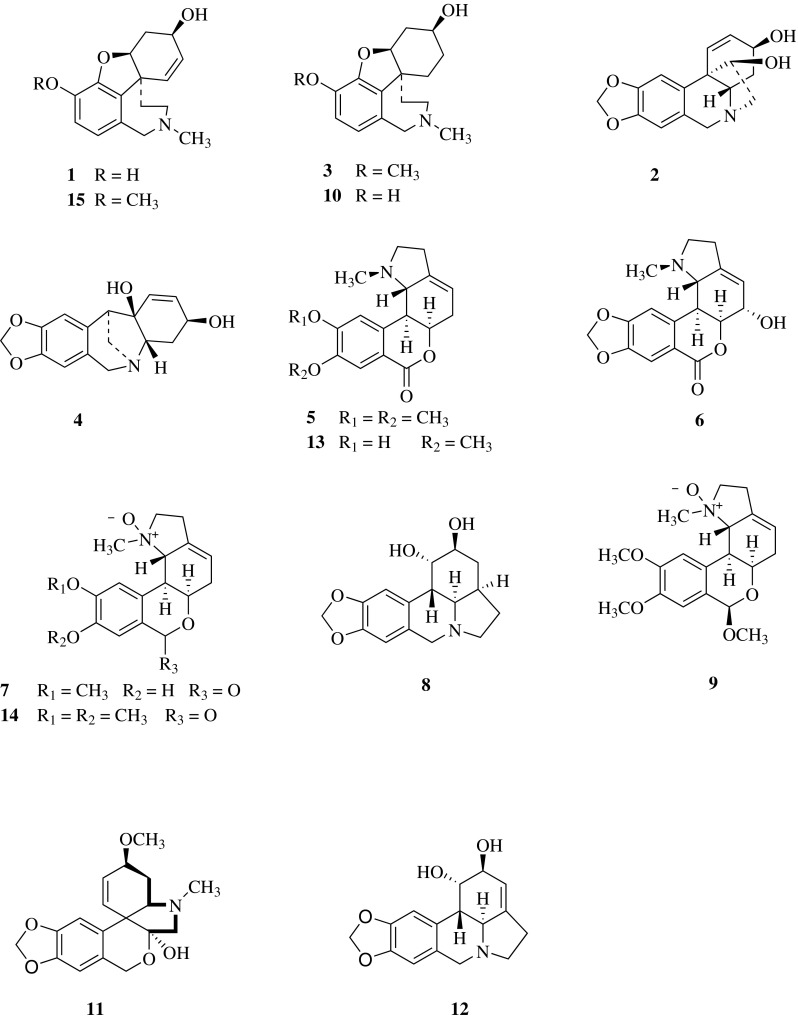
Structures of Compounds **1–15** from *Lycoris radiata*

The air-dried bulbs chips of *Lycoris radiata* were extracted with MeOH and separated by SiO_2_ CC, Sephadex LH-20 and HPLC to obtain 15 known compounds (Fig. 1). Structures of the compounds were identified by comparison of their MS and NMR data with those reported in the literature. They were respectively sanguinine (**1**) [15], 11-hydroxyvittatine (**2**) [16], lycoramine (**3**) [17], pancratinine C (**4**) [18], homolycorine (**5**) [19], hippeastrine (**6**) [20], *O*-demethylhomolycorine N-oxide (**7**) [21], zephyranthine (**8**) [22], *O*-methyllycorenine N-oxide (**9**) [23], lycoranine C (**10**) [24], tazettine (**11**) [25], lycorine (**12**) [26], 9-*O*-demethylhomolycorine (**13**) [27], homolycorine N-oxide (**14**) [23], galanthamine (**15**) [28].

### Anti-TMV Activity In Vivo

**Fig. 2:**
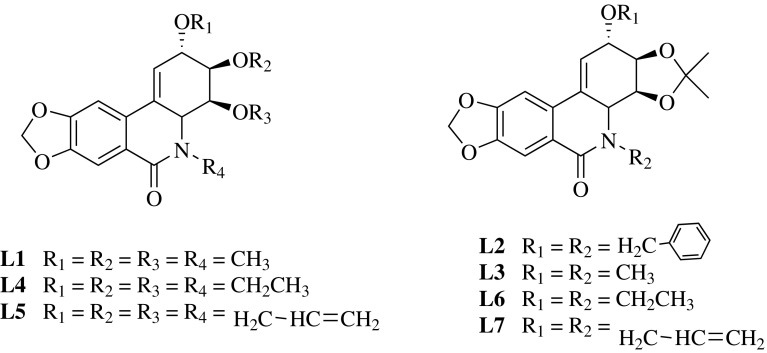
Structures of lycoricidine derivatives (**L1–L7**)

**Fig. 3:**
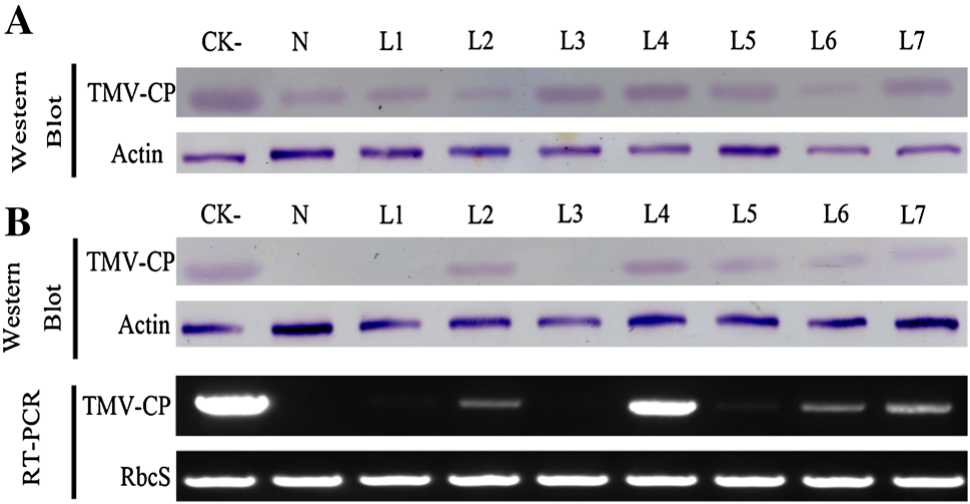
**a** Western-blot analysis of accumulation of TMV coat protein in the inoculated leaves treated with lycoricidine derivatives (**L1**–**L7**) (100 μg/mL) at 3 dpi. **b** Western-blot and RT-PCR analysis of accumulation of TMV coat protein in system leaves at 5 dpi. *CK*^*−*^ negative control, *N* ningnanmycin, Actin and RbcS serves as internal reference

The inhibitory activities of compounds **1**, **2**, **6**, **8**, **10**–**13** and lycoricidine derivatives (**L1**–**L7**) (Fig. 2) against TMV were tested by the half-leaf method and reported in Table 1, all of the compounds exhibited moderate inactivation effect. Lycoricidine derivatives showed better protective effect than curative effect, but both activities were stronger than the compounds from *Lycoris radiata.* Compounds **L1** (*N*-methyl-2,3,4-trimethoxylycoricidine) and **L3** (*N*-methyl-2-methoxy-3,4-acetonidelycoricidine) exhibited protective activities against TMV with the inhibitive rate of 60.8%, and 62.0%, comparable to that of the positive control Ningnanmycin (66.4%). Accordingly, it was worth to further study whether these lycoricidine derivatives still had same protective effects in the systemic infection host N. tabacum cv. K326. We carried out the assay in vivo along with western-blot to analyze TMV Coat Protein (CP) (Fig. 3). The result showed that in 3 days post inoculation (3 dpi), the TMV-CP accumulation in the inoculated leaves treated with compounds was less than that in the inoculated leaves of the negative control (Fig. 3a). The results of the system leaves (Fig. 3b) in 5 dpi TMV-CP was undetectable (L1 and L3) or relatively weaker (L5, L6 and L7) than that of the negative control. The above results indicated that the systemic infection of TMV was inhibited by the compounds. The order of inhibitory effect from strong to weak was Ningnanmycin, L1, L3, L5, L6, L7, L2 and L4.

**Table 1:** Anti-TMV activities on *N. glutinosa* in vivo

Compounds	Inhibition rate (%)		
	Inactivation effect	Protective effect	Curative effect
**L1**	52.3 ± 7	60.8 ± 7	45.5 ± 2
**L2**	63.3 ± 6	58.2 ± 2	52.7 ± 5
**L3**	54.7 ± 3	62.0 ± 3	39.9 ± 1
**L4**	60.0 ± 8	53.8 ± 3	52.2 ± 9
**L5**	55.3 ± 6	58.3 ± 6	44.5 ± 4
**L6**	15.0 ± 5	58.2 ± 5	50.2 ± 3
**L7**	59.3 ± 4	56.0 ± 2	52.1 ± 6
**1**	60.7 ± 8	–	33.3 ± 5
**2**	40.8 ± 10	27.6 ± 8	4.8 ± 5
**6**	41.2 ± 9	–	–
**8**	51.1 ± 7	19.5 ± 4	–
**10**	52.2 ± 9	43.1 ± 5	5.4 ± 3
**11**	42.4 ± 10	4.5 ± 3	–
**12**	52.2 ± 6	16.3 ± 7	22.6 ± 5
**13**	63.4 ± 2	6 ± 4	15.1 ± 7
Ningnanmycin	75.0 ± 9	66.4 ± 8	54.8 ± 3

The concentrations of compounds were 100μg/mL. All results are expressed as mean ± SD; n = 6 for all groups

To further confirm the anti-TMV efficiency at the transcription level of lycoricidine derivatives, semi-quantitative RT-PCR assay which is a highly sensitive and specific method was performed. According to the Western-blot assay of TMV-CP analysis, we chose the systemic leaves of *N. tabacum cv.* K326 at 5 dpi as materials. The result showed that in 5 dpi TMV-CP transcription levels in the systemic leaves of the compounds in the treatment group were less than that of  the negative control (Fig. 3b). And the compounds exhibited difference inhibitory effects on the systemic synthesis of TMV-CP. Specifically, the reducing orders of inhibitory effect were ningnanmycin, **L1**, **L3**, **L5**,** L6**, **L2**, **L7** and **L4**. It was agreed with the result of the western-blot analysis. From the above, Compounds **L1**, **L3** and **L5** exhibited anti-TMV activity through lower accumulation of both TMV-CP gene and TMV-CP protein. Among the lycoricidine derivatives, compounds **L1** and **L3** showed relatively higher induction activities on systemic leaves.

### Antiviral Activity Against TMV by Inducing Defensive Enzyme Activities in Systemic Leaves

**Fig. 4:**
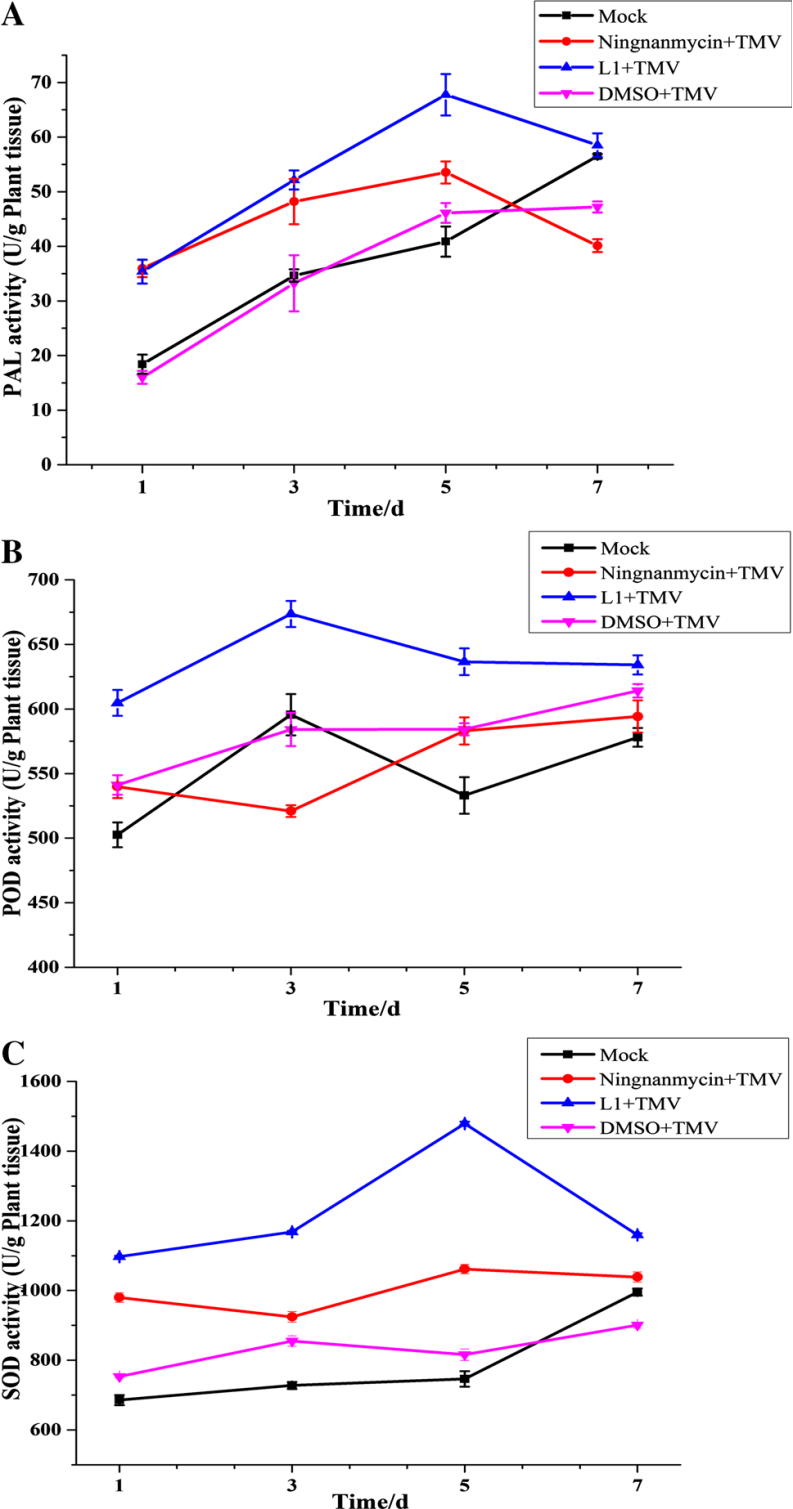
PAL (**a**), POD (**b**), and SOD (**c**) activity in tobacco plants treated with compound **L1** (100 μg/mL). mock, healthy tobacco; All results are expressed as mean ± SD; n = 3 for all groups

On the basis of the in vivo screening results, lycoricidine derivatives showed well protective effect. To the best of our knowledge, when plants were infected with various aggressive pathogens, to protect themselves, they developed various defensive responses, such as defense enzyme activity. Phenylalanine ammonia lyase (PAL), peroxidase (POD) and superoxide dismutase (SOD) are important defensive enzyme. PAL is a key regulatory enzyme in the biosynthesis of salicylic acid (SA) and establishment of SAR [29]. POD and SOD are antioxidant enzyme and play an important role in protecting plants from damage, they can effectively remove hydrogen peroxide, superoxide anion radical and other reactive oxygen from plant to reduce the damage of the membrane system and prevent lipid oxidation. Compound **L1** with good anti-TMV activity was chosen to study defensive enzyme activity. The data in Fig. 4 showed the variation of PAL, POD, and SOD activities under compound **L1** treatment. PAL participates in the biosynthesis of phenylpropanoids and can produce SA for defense against pathogens [30]. PAL activity of **L1** + TMV treatment group was higher than that of other treatments. PAL activity of ** L1** + TMV treatment group increased from day 1 to day 5 and reached the highest value on the fifth day (Fig. 4a). It’s common that POD activity can increase to improve the adaptability of plants when plants are subjected to exotic stress. The difference between POD activity of **L1** + TMV treatment group and that of other treatment was not significant (Fig. 4b), but **L1** + TMV treatment was still higher than others. The main function of SOD is to remove the superoxide anions in the plant, it’s general that the higher the SOD activity is, the stronger the resistance of plants produce. In compound **L1** treated groups, SOD activity of tobacco which was observably higher than others increased at day 1 to day 5, reached maximum on the fifth day, and dropped from day 5 to day 7 (Fig. 4c). These results suggested that compound **L1** could protect plants from pathogens, which is associated with elevated defensive enzyme activity.

In summary, 15 known amaryllidaceae alkaloids were isolated from the bulbs of *Lycoris radiata*. Some of the compounds and lycoricidine derivatives showed moderate inactivation effect. Lycoricidine derivatives showed potent protective effect. Compounds **L1**, **L3** and **L5** could reduce TMV-CP accumulation and gene transcription, especially compounds **L1** and **L3** exhibited relatively good protective effect. Defensive enzyme activity assay confirmed that compound **L1** could increase defensive enzyme activity to protect plants from pathogens. Lycoricidine alkaloids are found in Amaryllidaceae plant and Liliaceae plant [5]. Since there are abundant resources for getting lycoricidine alkaloids, perhaps lycoricidine alkaloids could be a good choice for anti-TMV agents.

## Experimental Section

### General Experimental Procedures

1D and 2D NMR were recorded on Bruker AM-400, Bruker DRX-500 or Avance III-600 spectrometers (Bruker, Bremerhaven, Germany). Mass spectra were run on a Waters HPLC-Thermo Finnigan LCQ Advantage ion trap mass spectrometer (Milford, PA). Silica gel (100–200 mesh, 200–300 mesh) for column chromatography and TLC plates (GF254) were obtained from Qingdao Haiyang Chemical Company (Haiyang, Qingdao, China). Sephadex LH-20 (40–70 μm) for column chromatography was purchased from Amersham Pharmacia Biotech AB (Uppsala, Sweden). HPLC was carried out on Agilent 1200 liquid chromatography system (Agilent Technologies, Santa Clara, CA, USA) equipped with diode array detector (DAD). Fractions were visualized by silica gel plates sprayed with dragendorff’s reagent. SDS-PAGE and Western-blot were carried out using a Bio-Rad electro transfer system. RT-PCR was measured by Bio-Rad T100™ PCR.

### Plant Material

The bulbs of *Lycoris radiata* were collected in Guiyang, Guizhou Province, in August 2012. The plant was identified by Assistant Professor Yu Chen. A voucher specimen (No. 1010725) has been deposited in the State Key Laboratory of Phytochemistry and Plant Resources in West China, Kunming Institute of Botany, CAS.

### Lycoricidine Derivatives

Lycoricidine derivatives (Fig. 2) were produced by our laboratory, according to the synthetic routes established by Chen et al. [12].

### Extraction and Isolation

The air-dried bulbs of *Lycoris radiata* (90 kg) were powdered and extracted three times with MeOH for 3 h under reflux. The crude extract was dissolved with 1.5% HCl solution. The aqueous phase was extracted with Petroleum ether (PE) and Ethyl acetate (EtOAc), then basified to pH 9–10 with ammonia (20%) and extracted with CHCl_3_ to obtain crude alkaloidal extract (813.6 g). The crude alkaloidal extract was performed on silica column chromatography, eluted with CHCl_3_–MeOH (1:0-0:1) to obtain five fractions (A–E). Fr. D (57.4 g) was further separated to obtain three subfractions (D1–D3) by SiO_2_ CC with CHCl_3_–MeOH (5:1–0:1). Fr. D1 (10.3 g) was subjected to SiO_2_ CC and eluted with CHCl_3_–MeOH (2:1–0:1) to yield compounds **1** (60 mg) and **2** (90 mg), and it was further purified by Sephadex LH-20 (CHCl_3_–MeOH, 1:1) to produce compounds **3** (14 mg), **7** (8 mg) and **8** (24 mg). Fr. D2 was applied to SiO_2_ CC with an elution of CHCl_3_–MeOH (1:2–0:1), and then purified by silica gel and HPLC to give compounds **4** (10 mg), **5** (4 mg), **6** (8 mg) and **9** (7 mg). Fr. E (40 g) was further separated to obtain four subfractions (E1–E4) by SiO_2_ CC with CHCl_3_–MeOH (3:1–0:1). Fr. E2 (15 g) was extensively chromatographed over columns of SiO_2_ and Sephadex LH-20 (CHCl_3_–MeOH, 1:1) to afford compounds **10** (32 mg), **11** (27 mg), **12** (24 mg) and **13** (36 mg). Fr. E3 (9.3 g) was applied to SiO_2_ CC with an eluent of CHCl_3_–MeOH (2:1) and further purified through HPLC (40% MeOH) to provide compounds **14** (6 mg) and **15** (26 mg).

### Anti-TMV Assays

#### Preparation of Screening Materials

TMV(Strain U1) was maintained and cultured in *Nicotiana tabacun cv.* K326. TMV particles were purified using Gooding’s method [31] and stored at − 20 °C for later use. The purified virus was diluted to 50 μg/mL with 0.01 M phosphate-buffered saline (PBS) before use.

*Nicotiana glutinosa* and *N. tabacum cv.* K326 plants were cultivated in an insect-free greenhouse. *N. glutinosa* was used as a local lesion host, and *N. tabacum cv. K326* was used to determine systemic TMV infection when the plants grew to the 5–6 leaf stage.

The tested compounds were dissolved in dimethyl sulfoxide (DMSO) and diluted with distilled H_2_O to the required concentrations. The solution of equal concentration of DMSO was used as negative control (CK^−^). Ningnanmycin, a commercial antiviral agent, was used as a positive control. The healthy plants were used as mock for assays with *N. tabacum cv.* K326.

#### Half-Leaf Method

Inactivation effect of compounds against TMV in vivo. The virus was inhibited by mixing it with the compound solution at the same volume for 30 min. The mixture was then inoculated on the left half side of one leaf of *N. glutinosa*, whereas the right half side of the leaves was inoculated with the mixture of solvent and the virus for control. The local lesion numbers were recorded 3–4 days after inoculation. The experiment was tested in triplicate with each compound.

##### Protective Effect of Compounds Against TMV In Vivo

Similarly grown *N. glutinosa* was selected at the 5–6 leaf stage, and 100 μg/mL compound solution was smeared on the whole fully open leaves. Solvent and Ningnanmycin which were smeared on other plants were used as negative control (CK^−^) and positive control, respectively. After 12 h, each leaf was mechanically inoculated with 200 μL of TMV at the concentration of 50 μg/mL. Then the leaves were washed with water once or twice. The local lesion numbers were recorded 3–4 days after inoculation. Three repetitions were conducted for each compound.

##### Curative Effect of Compounds Against TMV In Vivo

Growing leaves of *N. glutinosa* of the same ages were selected. TMV (concentration of 50 μg/mL) was inoculated on the whole leaves. Then the leaves were washed with water and dried. 12 h later, the compound solution (100 μg/mL) was smeared on the whole fully open leaves. Solvent and Ningnanmycin which were smeared on other plants were used as negative control (CK^−^) and positive control, respectively. The local lesion numbers were recorded 3–4 days after inoculation. Each compound was tested in triplicate.

##### Calculation of the Rate of TMV Inhibition

The inhibition rate of the compound was then calculated according to the following formula

$${\text{Inhibition rate }}\left( \% \right) = [(C - T)/C] \times 100\%$$

where C is the average number of local lesions of the negative control and T is the average number of local lesions of the treatment.

#### Effect of Compounds on Systemic Infection of TMV

Five pots of healthy *N. tabacum cv.* K326 plants at 5–6 leaf stage were chosen for the screening of one compound. The compound solution (100 μg/mL) was smeared on the whole plant. Solvent and Ningnanmycin were used as negative and positive controls, respectively. The leaves were inoculated with the TMV (the third to fourth leaves from the top) of one plant after 12 h and cultivated in an insect-free greenhouse. Three treatments were adopted: DMSO + TMV, Ningnanmycin + MV, and compounds + TMV. Tissue samples were collected at 3 and 5 days after inoculation treatment for western-blot assays to detect the TMV-CP, PCR analysis to detect the TMV-CP genes.

SDS-PAGE and Western Blot Analysis of TMV-CP. SDS-PAGE was performed as described previously [32]. Briefly, the tissue samples (0.1 g) were ground in protein loading buffer (40 g/L SDS, 10 mL/L β-ME, 200 mL/L glycerin, 2 g/L bromophenol blue, 0.1 mol/L Tris–HCl, pH 6.8), and then 3 μL of sample and 5 μL of marker (Blue Plus Protein Marker, 14–100 kDa) were loaded on a polyacrylamide gel (5% stacking gel, 12.5% separating gel). Samples were run in duplicate. After SDS-PAGE, the protein was transferred onto nitrocellulose membrane (0.22 μm) at 10 V for 30 min using an electrotransfer system (Bio-Rad Trans-Blot SD Semi-Dry Electrophoretic Transfer cell). The membrane was washed in TBST (1 mol/L Tris–HCl, pH 7.5; 1 mol/L NaCl; 0.05% Tween-20) and blocked with 5% nonfat milk powder in TBST at 37 °C for 1 h. The membrane was washed three times for 15 min with TBST and reacted with a mixture of 1:8000 polyclonal antibodies of TMV-CP and 1:3000 monoclonal antibodies of β-actin at 37 °C for 1 h, respectively. Then after washing the membrane three times for 15 min with TBST, 1:8000 alkaline phosphatase-conjugated antirabbit IgG (Sigma, St. Louis, MO) and 1:8000 alkaline phosphatase-conjugated antimouse IgG (Sigma, St. Louis, MO) were poured on the membrane to react at 37 °C for 1 h, respectively. After they were washed three times for 15 min with TBST, the membrane was incubated in substrate buffer (1 mol/L Tris–HCl, pH 9.5; 1 mol/L NaCl; 1 mol/L MgCl) with 330 μL/mL NBT and 165 μL/mL BCIP for 3–5 min in the dark until the bands of the CP were showed.

##### PCR Analysis of TMV-CP Genes

Total RNA was extracted from tobacco leaves (0.3 g, fresh weight) using TriPure Isolation Reagent (RNAiso Plus, TaKaRa, Japan) according to the manufacturer’s directions. The concentration of each RNA sample was measured with a NanoDrop ND-2000 spectrophotometer (NanoDrop Technologies, Wilmington, DE, USA). The integrity of the RNA sample was assessed by agarose gel electrophoresis. Total RNA (1 μg) was used for template of first-strand cDNA synthesis using extent reverse transcription kit (PrimeScript RT reagent Kit with gDNA Eraser, TaKaRa, Japan). Reverse transcription was carried out at 37 °C for 15 min and then at 85 °C for 5 s. The single-stranded DNA mixture was used as template in PCR. The primers for RT-PCR amplification were shown in Table 2. RT-PCR was conducted with 2 × EasyTaq PCR SuperMix (+ dye) (Transgen, Beijing, China). Each reaction volume (20 μL) consisted of 1 μL of 10 μM forward primer and reverse primer, respectively; 10 μL of 2 × EasyTaq PCR SuperMix (+ dye); 2 μL of cDNA and 6 μL of RNAse-free H_2_O. PCR amplification steps consisted of preliminary denaturation step at 95 °C for 3 min, followed by 34 cycles at 95 °C for 15 s, at 57 °C for 15 s, and at 72 °C for 1 min. PCR products were separated on 1% agarose gel in 1 × TAE buffer and visualized under UV light after staining with GelStain (Transgen, Beijing, China).

**Table 2:** Sequences of gene-specific primers used in RT-PCR analysis

Gene	Primers
TMV-CP-F	5′-GTTGATGAGTTCATGGAAGATG-3′
TMV-CP-R	5′-CAACCCTTCGATTTAAGTGGAG-3′
RbcS^a^-F	5′-CCTCTGCAGTTGCCACC-3′
RbcS-R	5′-CCTGTGGGTATGCCTTCTTC-3′

^a^RbcS, the small subunits of ribulose 1,5-bisphosphate carboxylase/oxygenase serves as internal reference gene

#### Determination of Defensive Enzyme Activities Assay

*N. tabacum* cv. K326 plants were sprayed with 100 μg/mL lycoricidine derivatives and solution of equal concentration of DMSO was used as negative control. Leaves were harvested 1, 3, 5, and 7 days respectively after spraying and used for quantitation of the phenylalanine ammonia lyase (PAL), peroxidase (POD) and superoxide dismutase (SOD) activities. Activities of PAL, POD and SOD were measured and calculated with enzyme assay reagent kits in accordance with manufacturer’s instructions (Nanjing Jiancheng Bioengineering Research Institute, China).

## Electronic supplementary material

Below is the link to the electronic supplementary material.

Supplementary material 1 (DOC 1511 kb)
