# Supplementary material for: Anti-TMV Effects of Amaryllidaceae Alkaloids Isolated from the Bulbs of Lycoris radiata and Lycoricidine Derivatives
Source: Nat Prod Bioprospect. 2018 May 18;8(3):189–97. doi: 10.1007/s13659-018-0163-0 (PMC5971033; doi:10.1007/s13659-018-0163-0)
Supplement: Supplementary file 1 — Supplementary material 1 (DOC 1511 kb) [file 13659_2018_163_MOESM1_ESM.doc]

Antiviral Effects of Amaryllidaceous Alkaloids Isolated from *Lycoris radiata* and Lycoricidine Derivatives

Dong-Qiong Yang ^a, b^, Zhao-Rong Chen ^c^, Duo-Zhi Chen ^a^, Xiao-Jiang Hao ^a^, Shun-Lin Li^a*^

🖂Shun-Lin Li

lisl@mail.kib.ac.cn

^a^State Key Laboratory of Phytochemistry and Plant Resources in West China, Kunming Institute of Botany, Chinese Academy of Sciences, Kunming 650201, People’s Republic of China.

^b^University of Chinese Academy of Sciences, Beijing 100049, People’s Republic of China

^c^Tianjin Agricultural University, Tianjin 300380, People’s Republic of China

**__________________________________**

**[*](http://pubs.acs.org/doi/full/10.1021/np200076t#cor1)**To whom correspondence should be addressed. Tel: +86-871-65223263; Fax: +86-871-65223070; E-mail: lisl@mail.kib.ac.cn

**Table of contents**

Figure S1. ^1^H NMR spectrum of Sanguinine (1) in CDCl_3_….............................................4

Figure S2. ^1^C NMR spectrum of Sanguinine (1) in CDCl_3_….............................................4

Figure S3. MS spectrum of Sanguinine (1) in CDCl_3_….......................................................5

Figure S4. ^1^H NMR spectrum of 11-Hydroxyvittatine (2) in MeOD…............................5

Figure S5. ^1^C NMR spectrum of 11-Hydroxyvittatine (2) in MeOD…............................6

Figure S6. MS spectrum of 11-Hydroxyvittatine (2) in MeOD….....................................6

Figure S7. ^1^H NMR spectrum of Lycoramine (3) in CDCl_3_…...........................................7

Figure S8. ^1^C NMR spectrum of Lycoramine (3) in CDCl_3_…...........................................7

Figure S9. MS spectrum of Lycoramine (3) in CDCl_3_….....................................................8

Figure S10. ^1^H NMR spectrum of Pancratinine C (4) in MeOD…...................................8

Figure S11. ^1^C NMR spectrum of Pancratinine C (4) in MeOD…....................................9

Figure S12. MS spectrum of Pancratinine C (4) in MeOD…..............................................9

Figure S13. ^1^H NMR spectrum of Homolycorine (5) in MeOD…...................................10

Figure S14. ^1^C NMR spectrum of Homolycorine (5) in MeOD…...................................10

Figure S15. MS spectrum of Homolycorine (5) in MeOD….............................................11

Figure S16. ^1^H NMR spectrum of Hippeastrine (6) in MeOD…......................................11

Figure S17. ^1^C NMR spectrum of Hippeastrine (6) in MeOD…......................................12

Figure S18. MS spectrum of Hippeastrine (6) in MeOD…...............................................12

Figure S19. ^1^H NMR spectrum of O-Demethylhomolycorine N-oxide (7) in MeOD.13

Figure S20. ^1^C NMR spectrum of O-Demethylhomolycorine N-oxide (7) in MeOD..13

Figure S21. MS spectrum of O-Demethylhomolycorine N-oxide (7) in MeOD…........................................................................................................................................14

Figure S22. ^1^H NMR spectrum of Zephyranthine (8) in MeOD…..................................14

Figure S23. ^1^C NMR spectrum of Zephyranthine (8) in MeOD…...................................15

Figure S24. MS spectrum of Zephyranthine (8) in MeOD….............................................15

Figure S25. ^1^H NMR spectrum of O-Methyllycorenine N-oxide (9) in CDCl_3_….........16

Figure S26. ^1^C NMR spectrum of O-Methyllycorenine N-oxide (9) in CDCl_3_…........16

Figure S27. MS spectrum of O-Methyllycorenine N-oxide (9) in CDCl_3_…...................17

Figure S28. ^1^H NMR spectrum of Lycoranine C (10) in MeOD…...................................17

Figure S29. ^1^C NMR spectrum of Lycoranine C (10) in MeOD…..................................18

Figure S30. MS spectrum of Lycoranine C (10) in MeOD…............................................18

Figure S31. ^1^H NMR spectrum of Tazettine (11) in CDCl_3_…...........................................19

Figure S32. ^1^C NMR spectrum of Tazettine (11) in CDCl_3_…...........................................19

Figure S33. MS spectrum of Tazettine (11) in CDCl_3_….....................................................20

Figure S34. ^1^H NMR spectrum of Lycorine (12) in CDCl_3_…............................................20

Figure S35. ^1^C NMR spectrum of Lycorine (12) in CDCl_3_…............................................21

Figure S36. MS spectrum of Lycorine (12) in CDCl_3_…......................................................21

Figure S37. ^1^H NMR spectrum of 9-O-Demethylhomolycorine (13) in MeOD…........22

Figure S38. ^1^C NMR spectrum of 9-O-Demethylhomolycorine (13) in MeOD…........22

Figure S39. MS spectrum of 9-O-Demethylhomolycorine (13) in MeOD….................23

Figure S40. ^1^H NMR spectrum of Homolycorine N-oxide (14) in MeOD….................23

Figure S41. ^1^C NMR spectrum of Homolycorine N-oxide (14) in MeOD….................24

Figure S42. MS spectrum of Homolycorine N-oxide (14) in MeOD…...........................24

Figure S43. ^1^H NMR spectrum of Galanthamine (15) in CDCl_3_…..................................25

Figure S44. ^1^C NMR spectrum of Galanthamine (15) in CDCl_3_…..................................25

Figure S45. MS spectrum of Galanthamine (15) in CDCl_3_…...........................................26


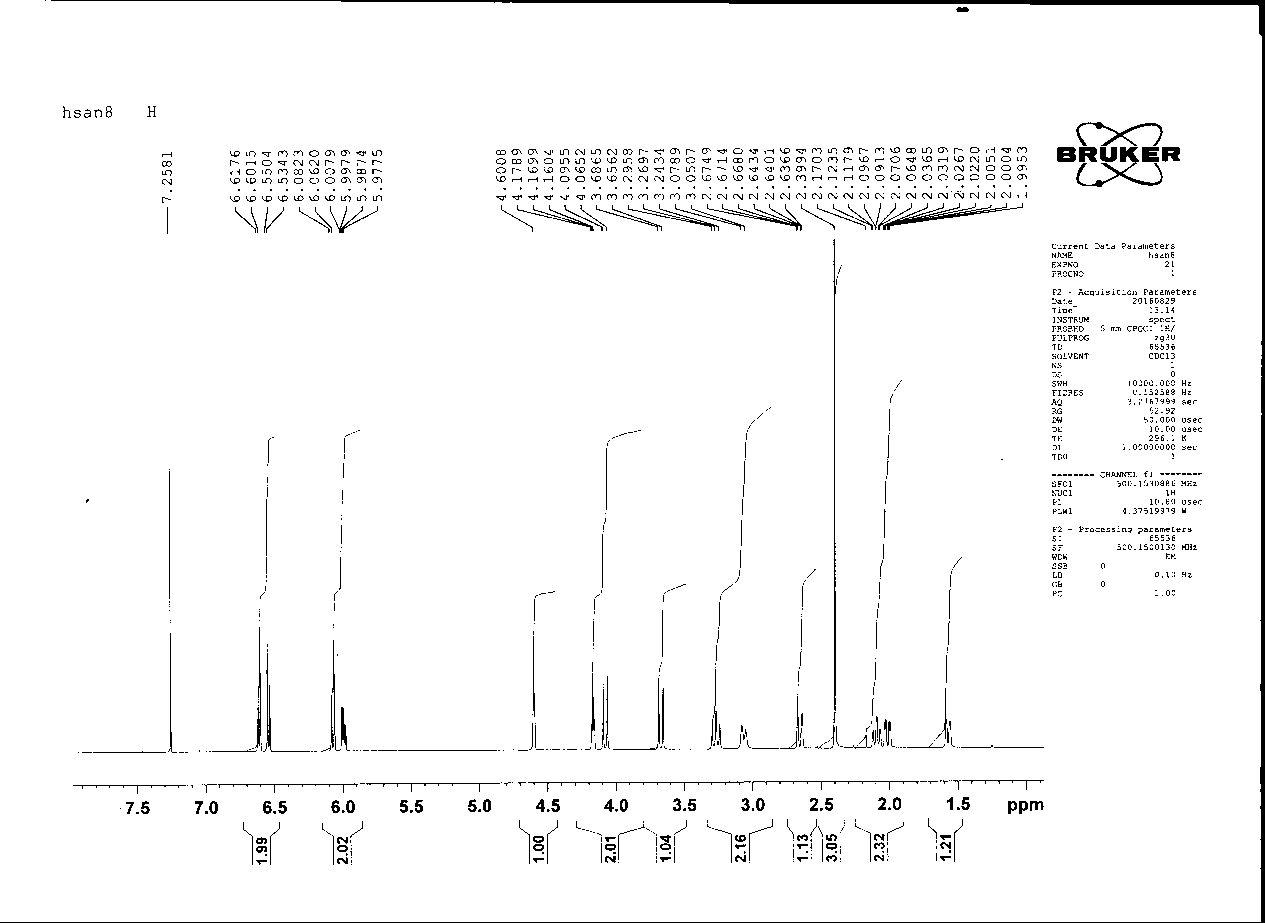


Figure S1. ^1^H NMR spectrum of Sanguinine (1) in CDCl_3_


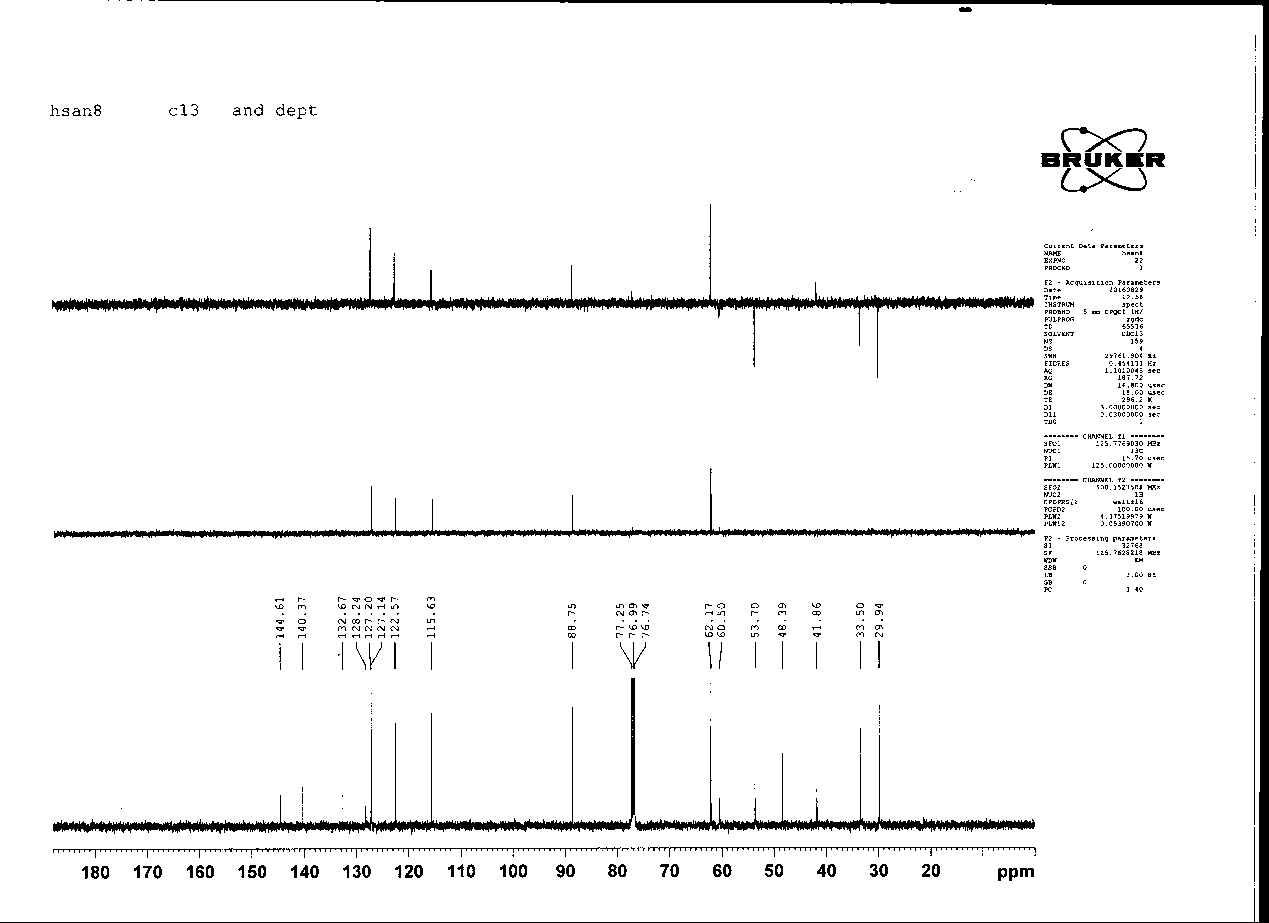


Figure S2. ^1^C NMR spectrum of Sanguinine (1) in CDCl_3_


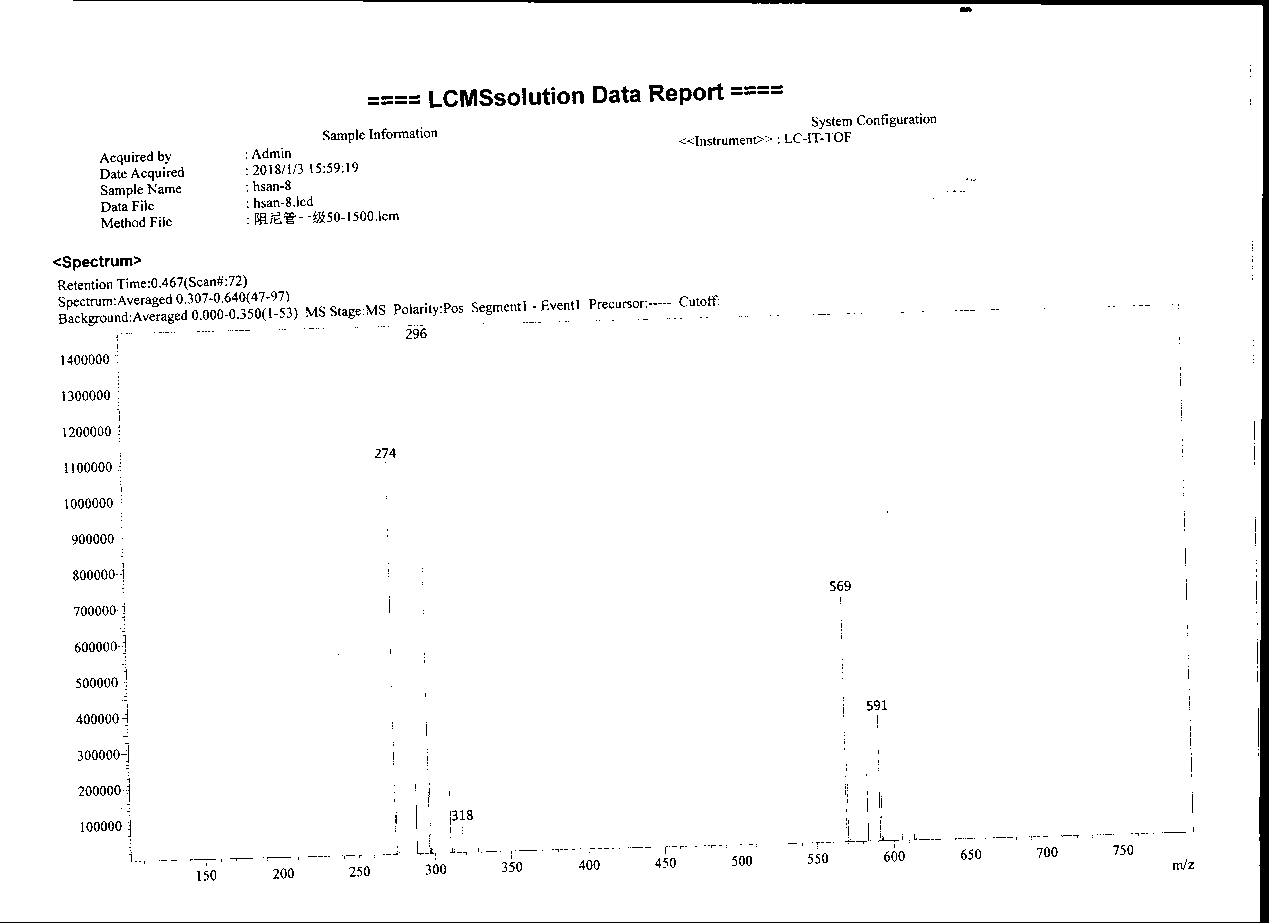


Figure S3. MS spectrum of Sanguinine (1) in CDCl_3_


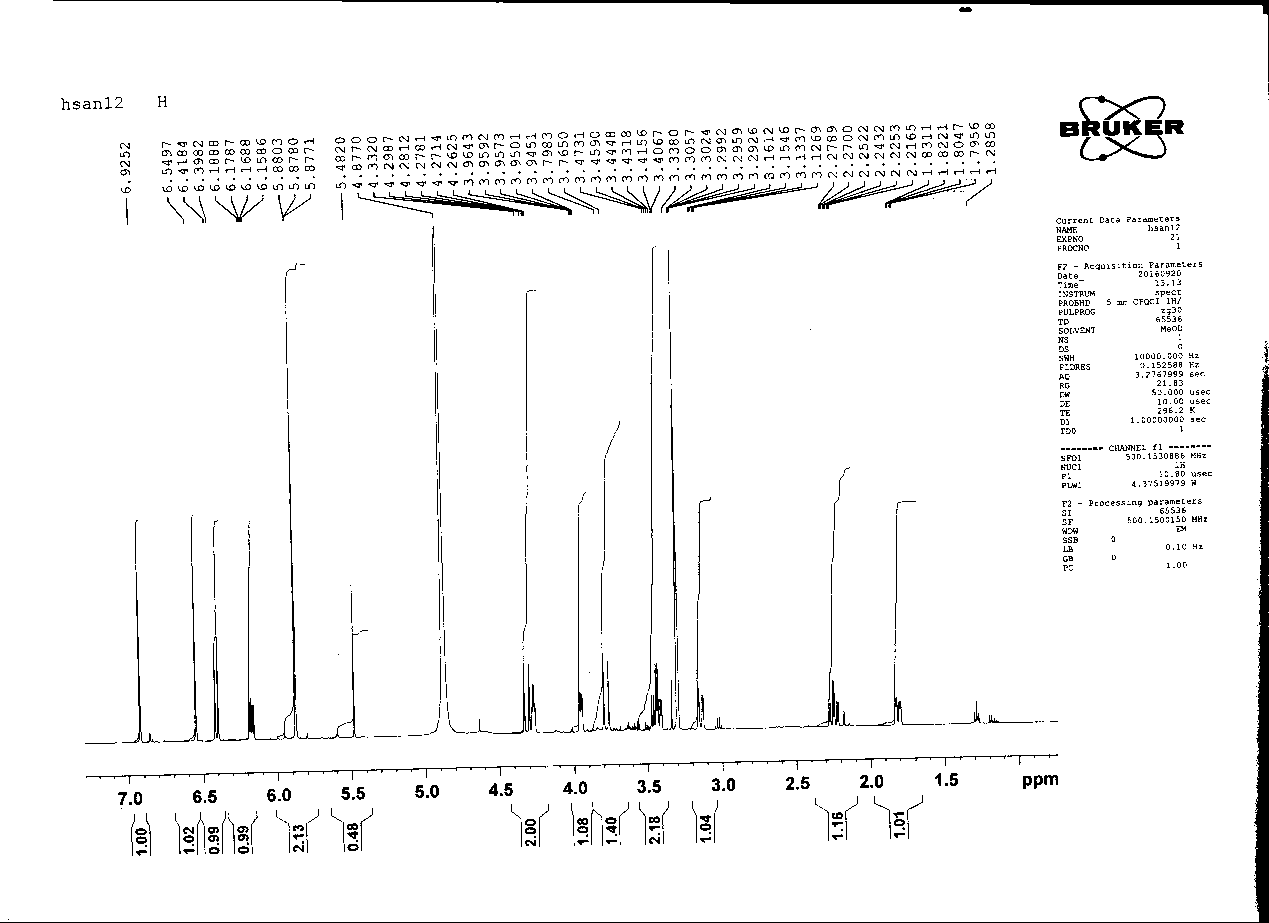


Figure S4. ^1^H NMR spectrum of 11-Hydroxyvittatine (2) in MeOD


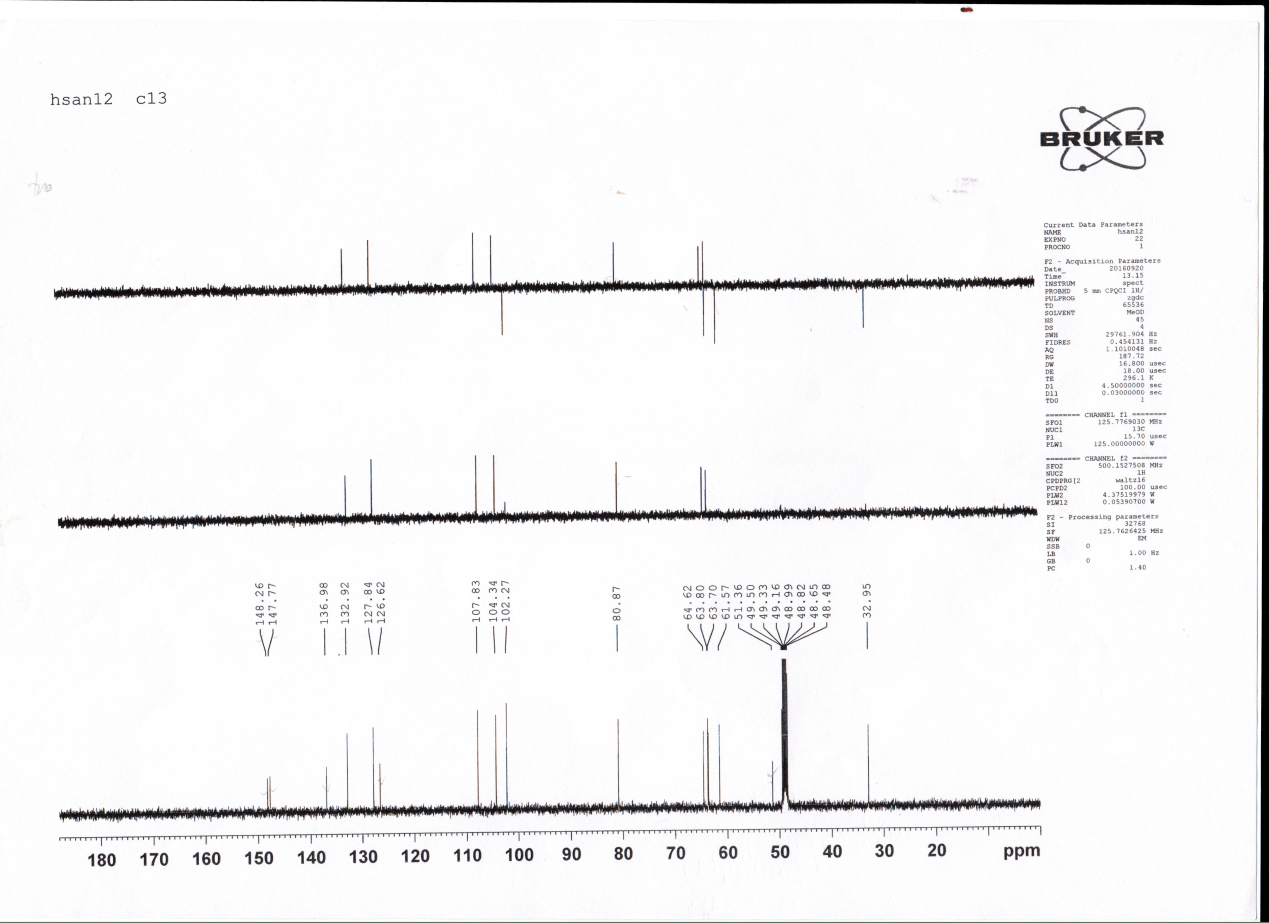


Figure S5. ^1^C NMR spectrum of 11-Hydroxyvittatine (2) in MeOD


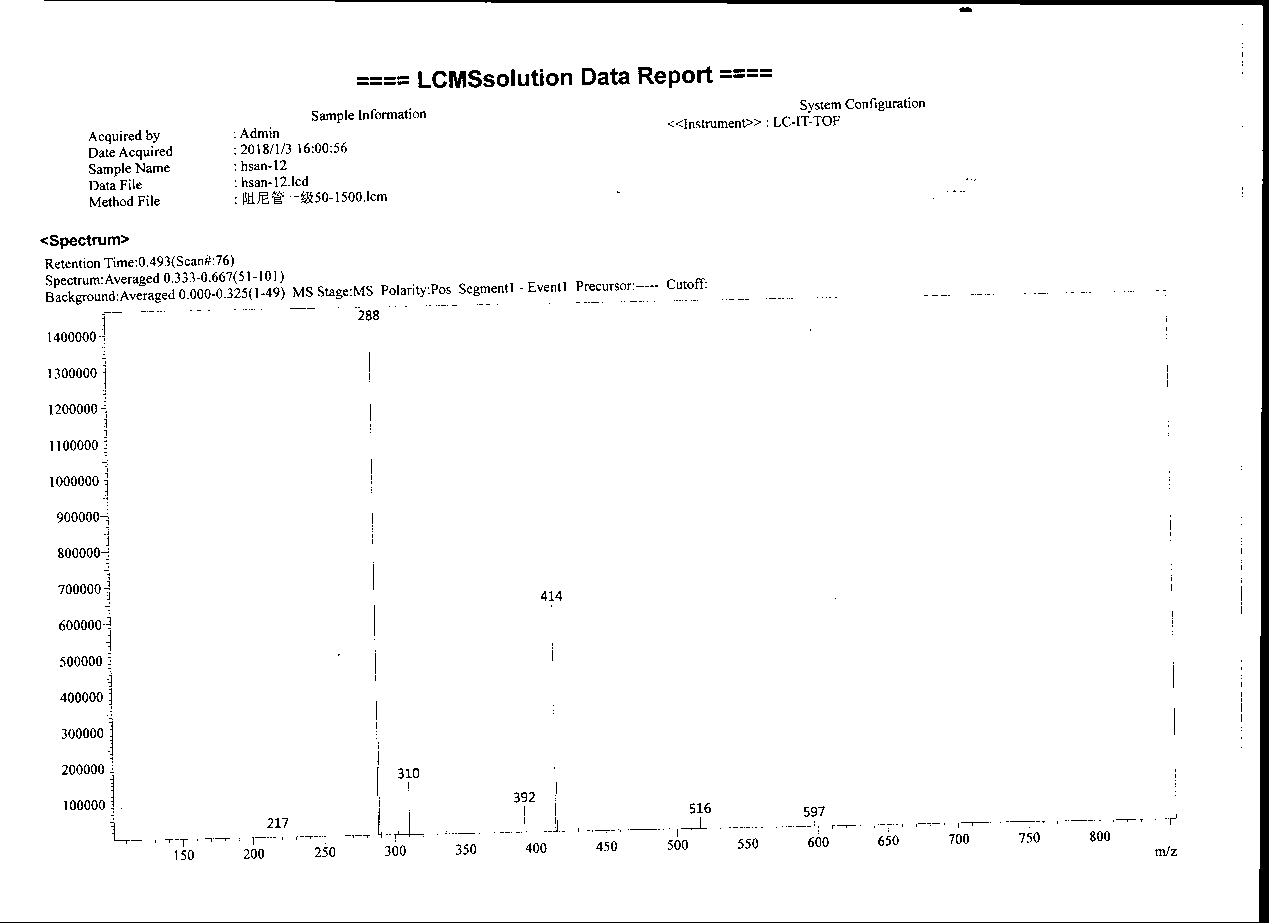


Figure S6. MS spectrum of 11-Hydroxyvittatine (2) in MeOD


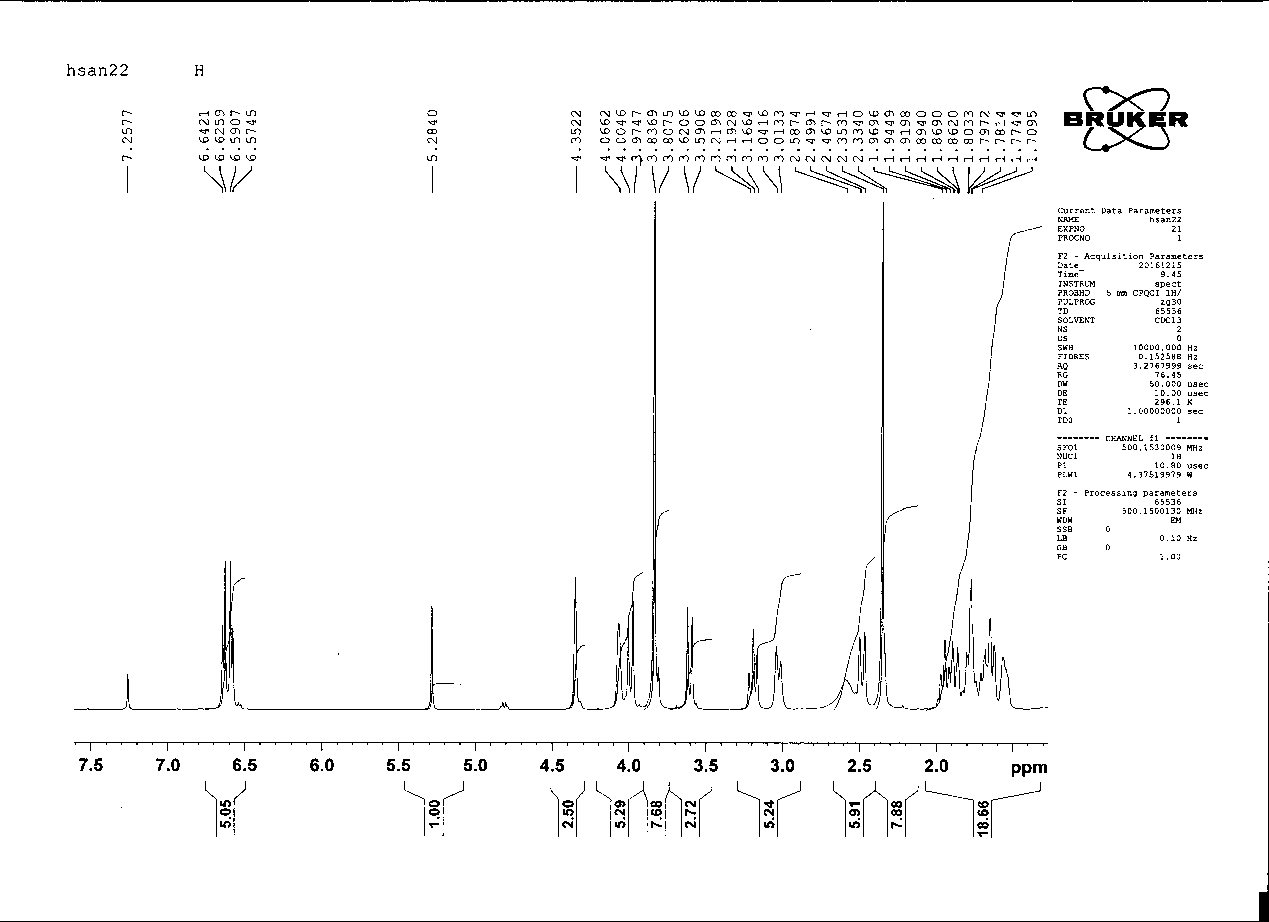


Figure S7. ^1^H NMR spectrum of Lycoramine (3) in CDCl_3_


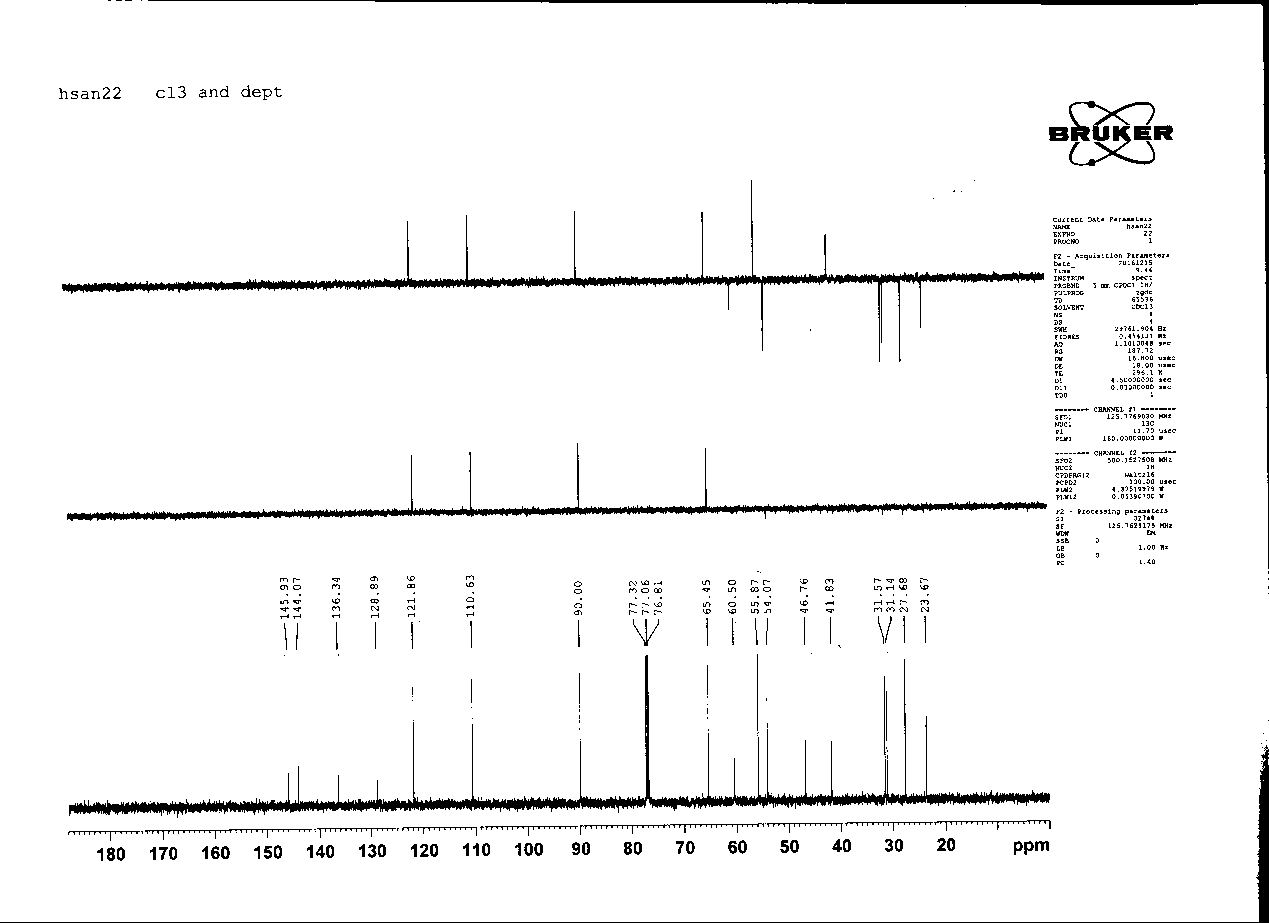


Figure S8. ^1^C NMR spectrum of Lycoramine (3) in CDCl_3_


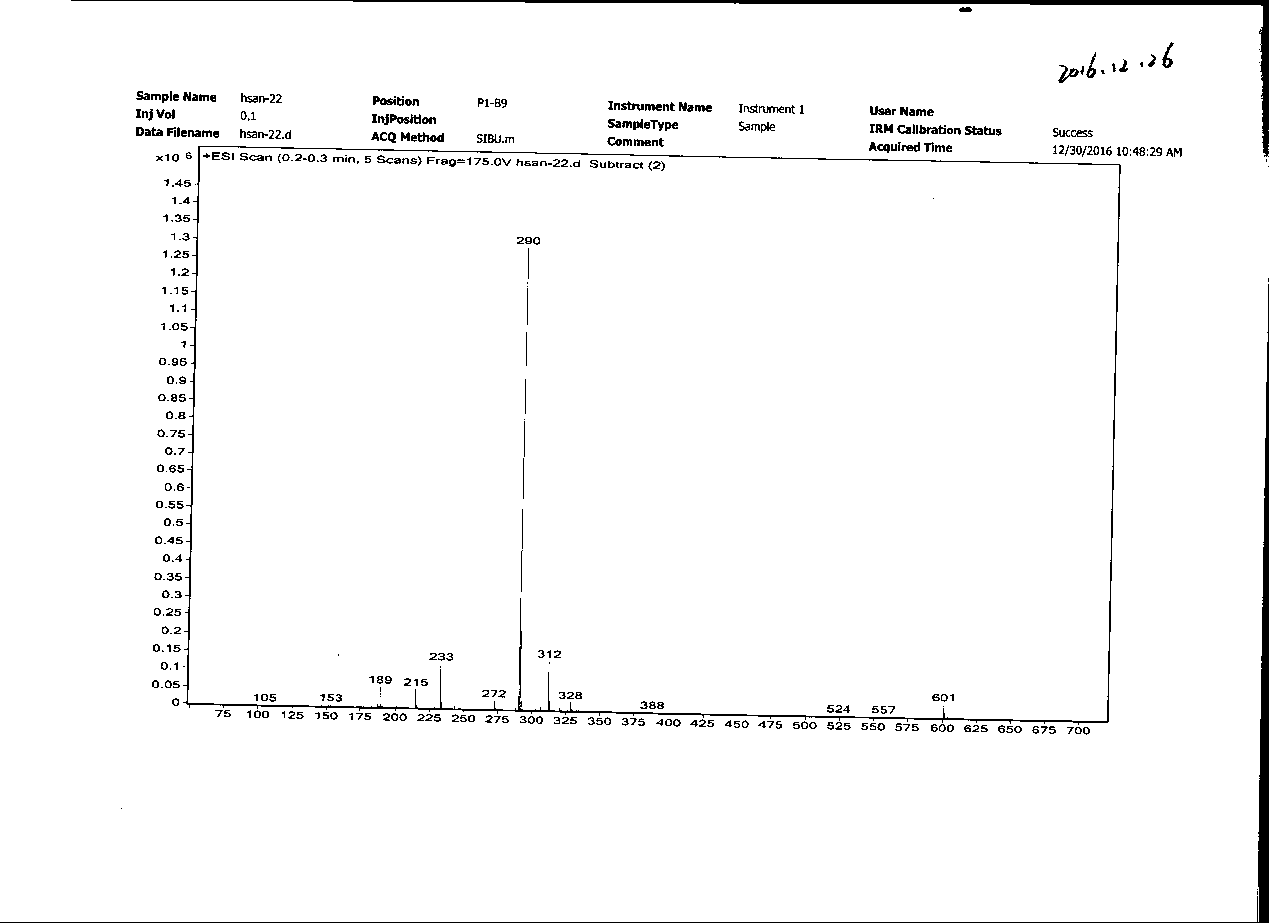


Figure S9. MS spectrum of Lycoramine (3) in CDCl_3_


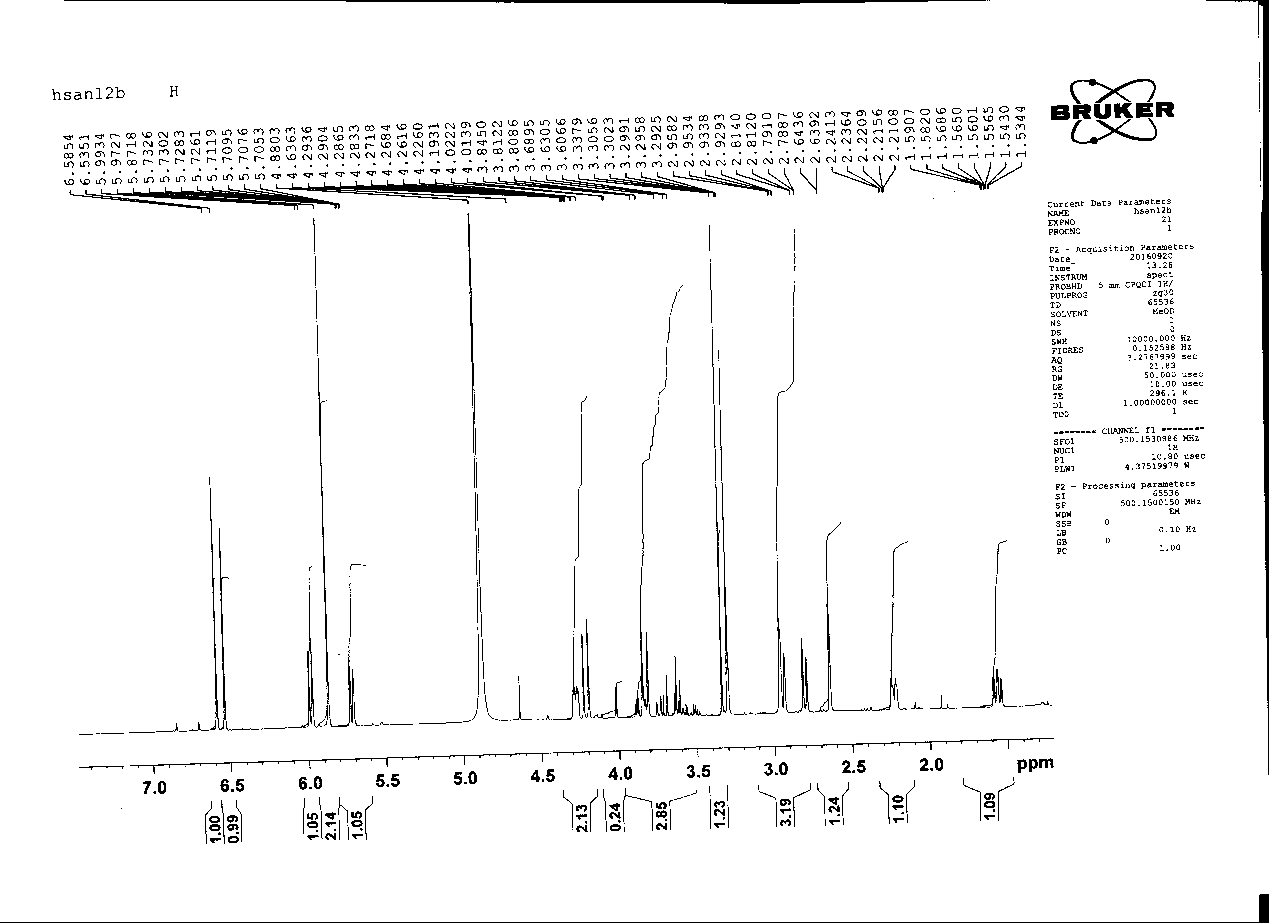


Figure S10. ^1^H NMR spectrum of Pancratinine C (4) in MeOD


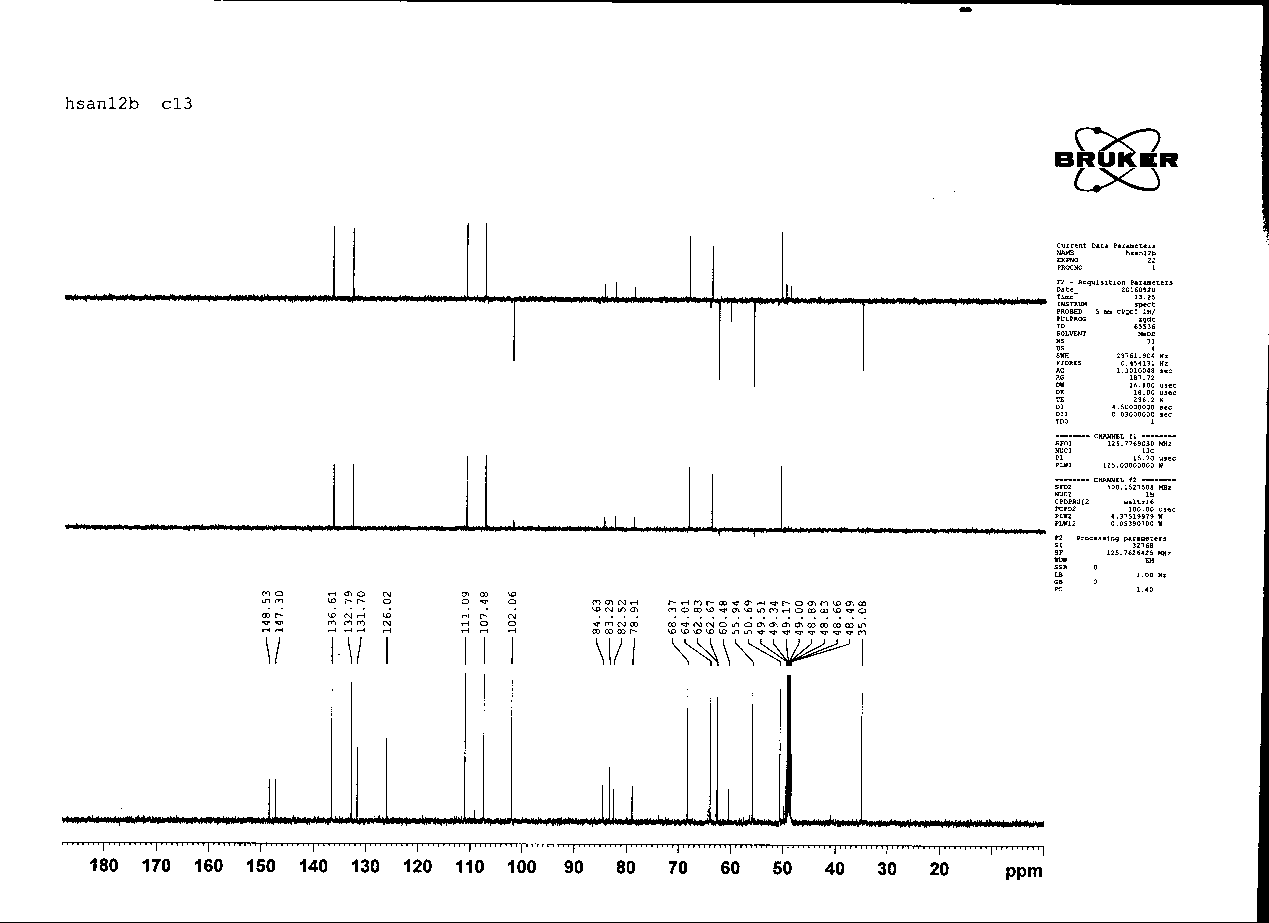


Figure S11. ^1^C NMR spectrum of Pancratinine C (4) in MeOD


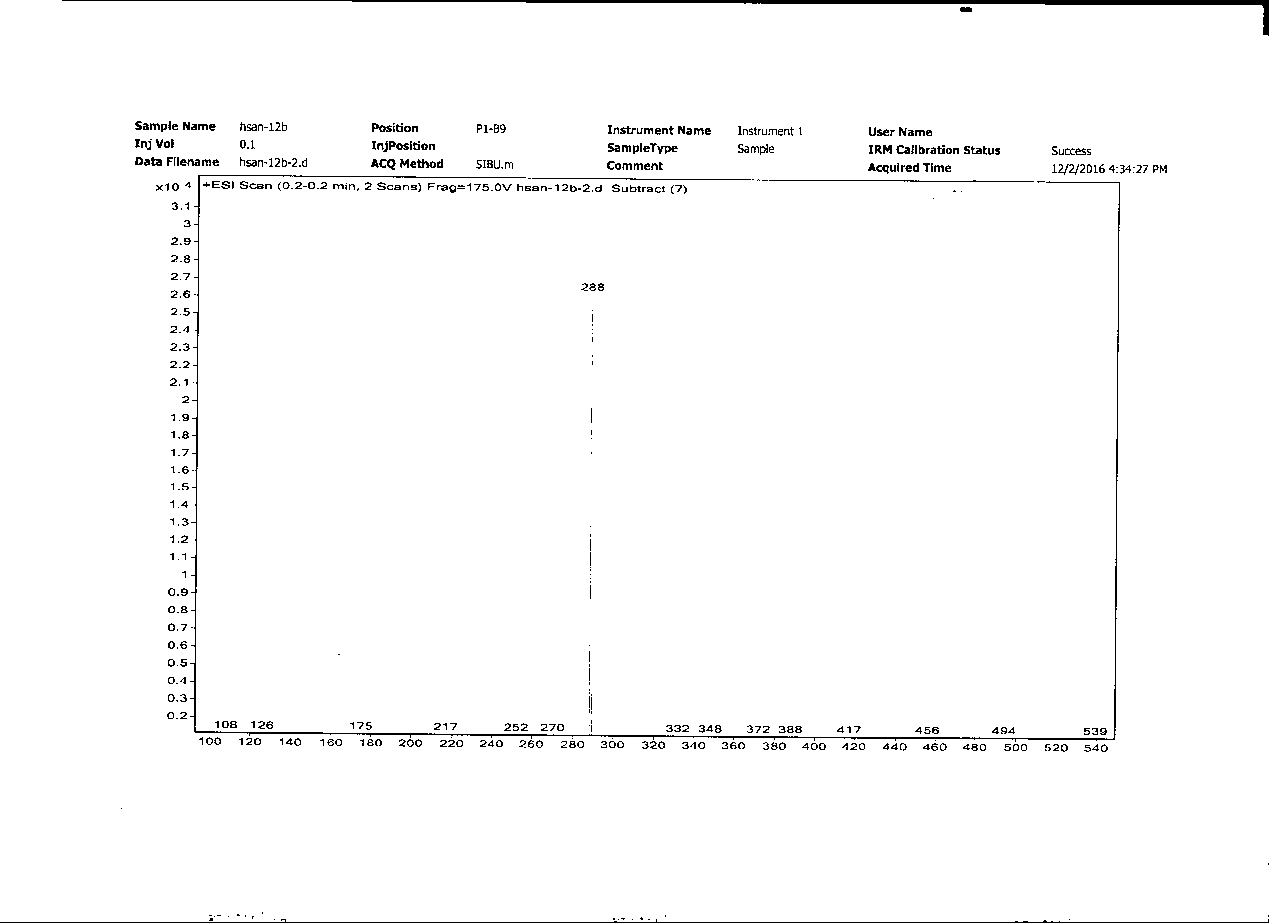


Figure S12. MS spectrum of Pancratinine C (4) in MeOD


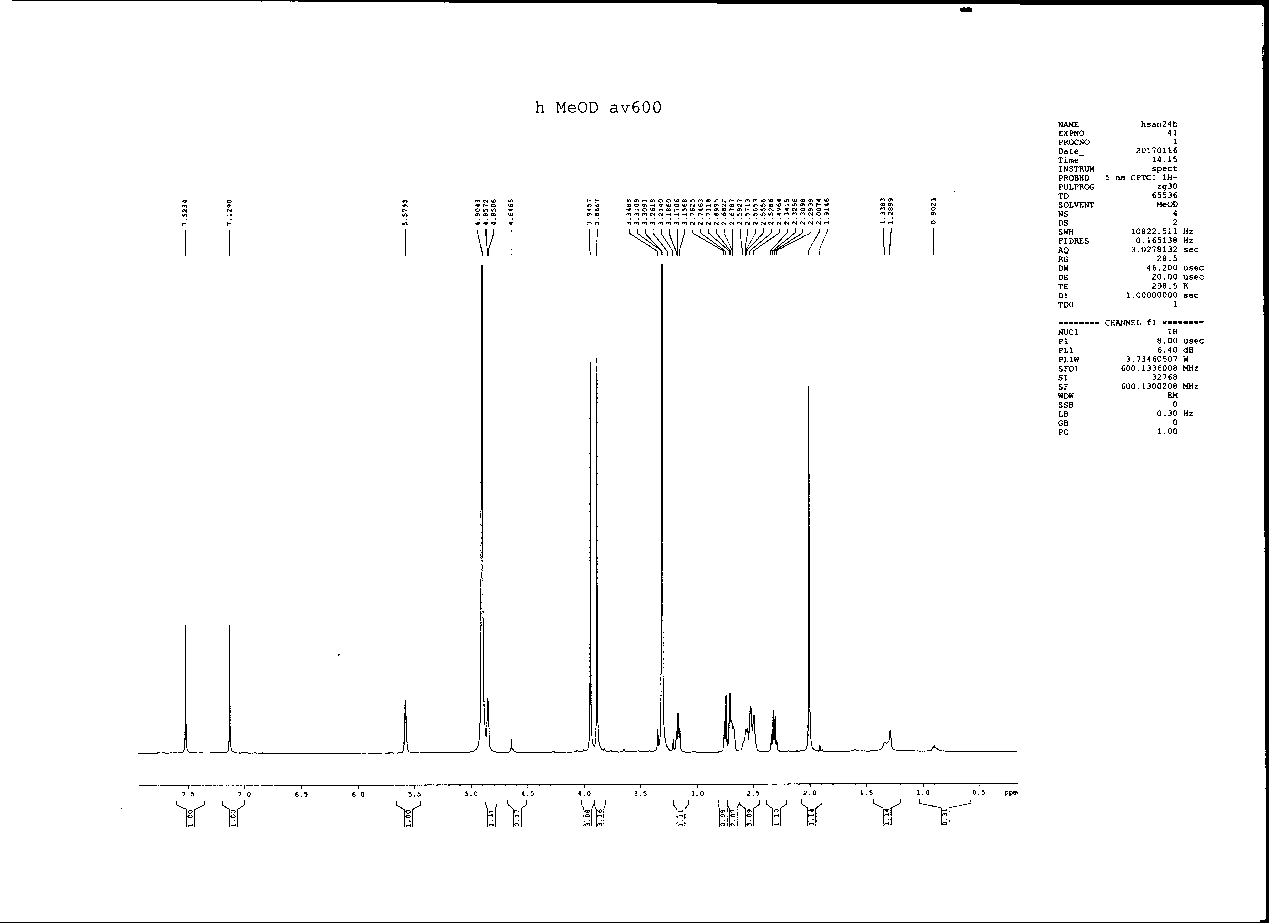


Figure S13. ^1^H NMR spectrum of Homolycorine (5) in MeOD


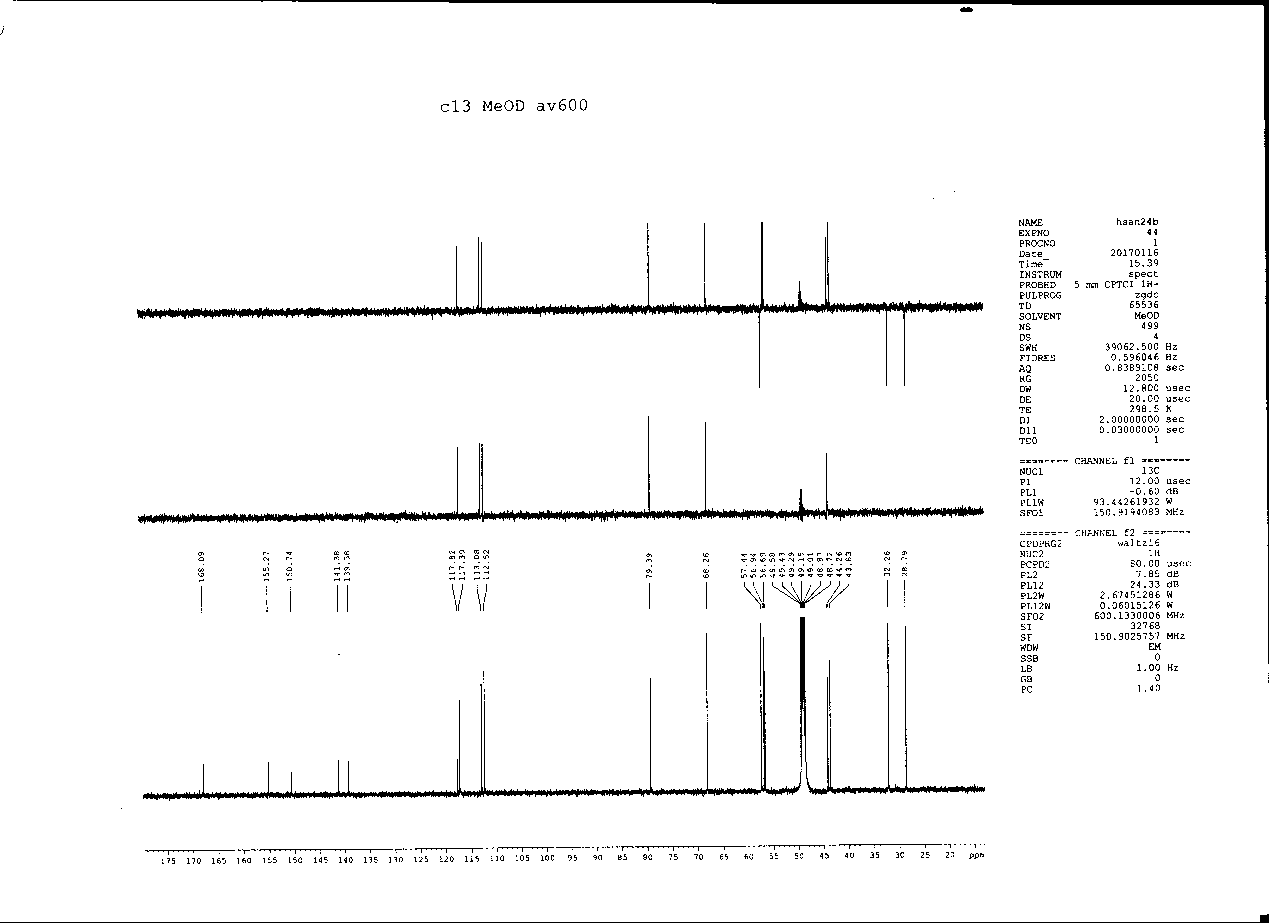


Figure S14. ^1^C NMR spectrum of Homolycorine (5) in MeOD


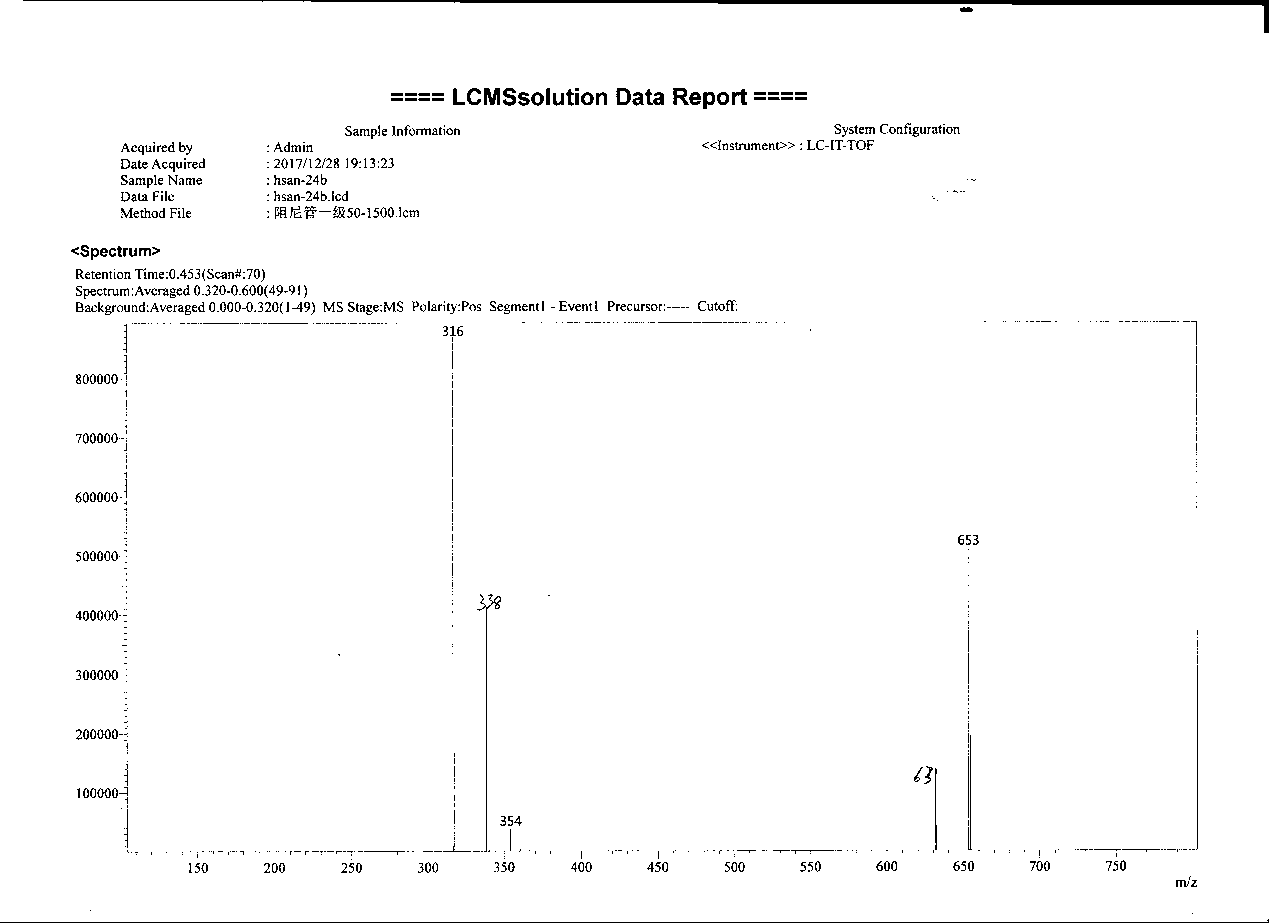


Figure S15. MS spectrum of Homolycorine (5) in MeOD


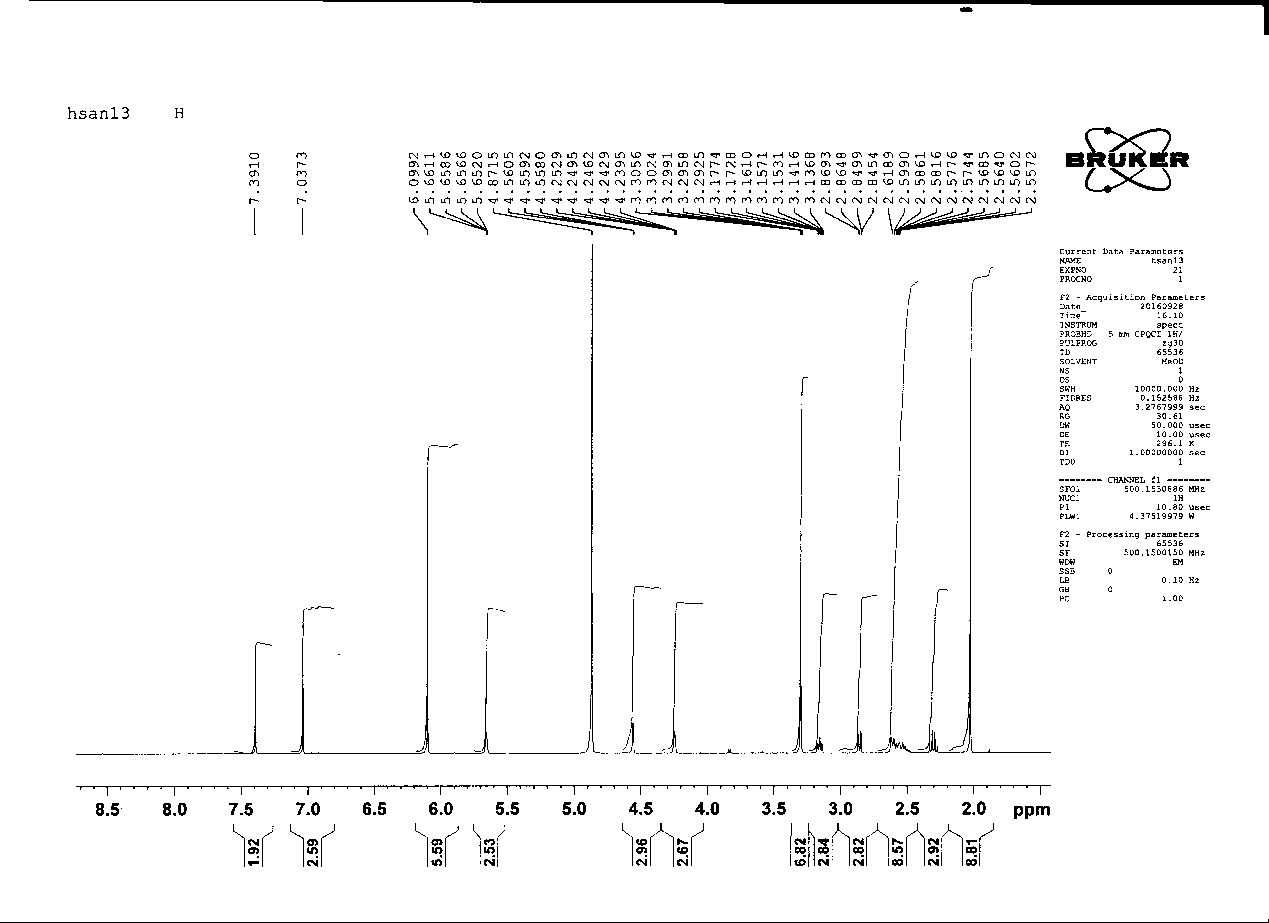


Figure S16. ^1^H NMR spectrum of Hippeastrine (6) in MeOD


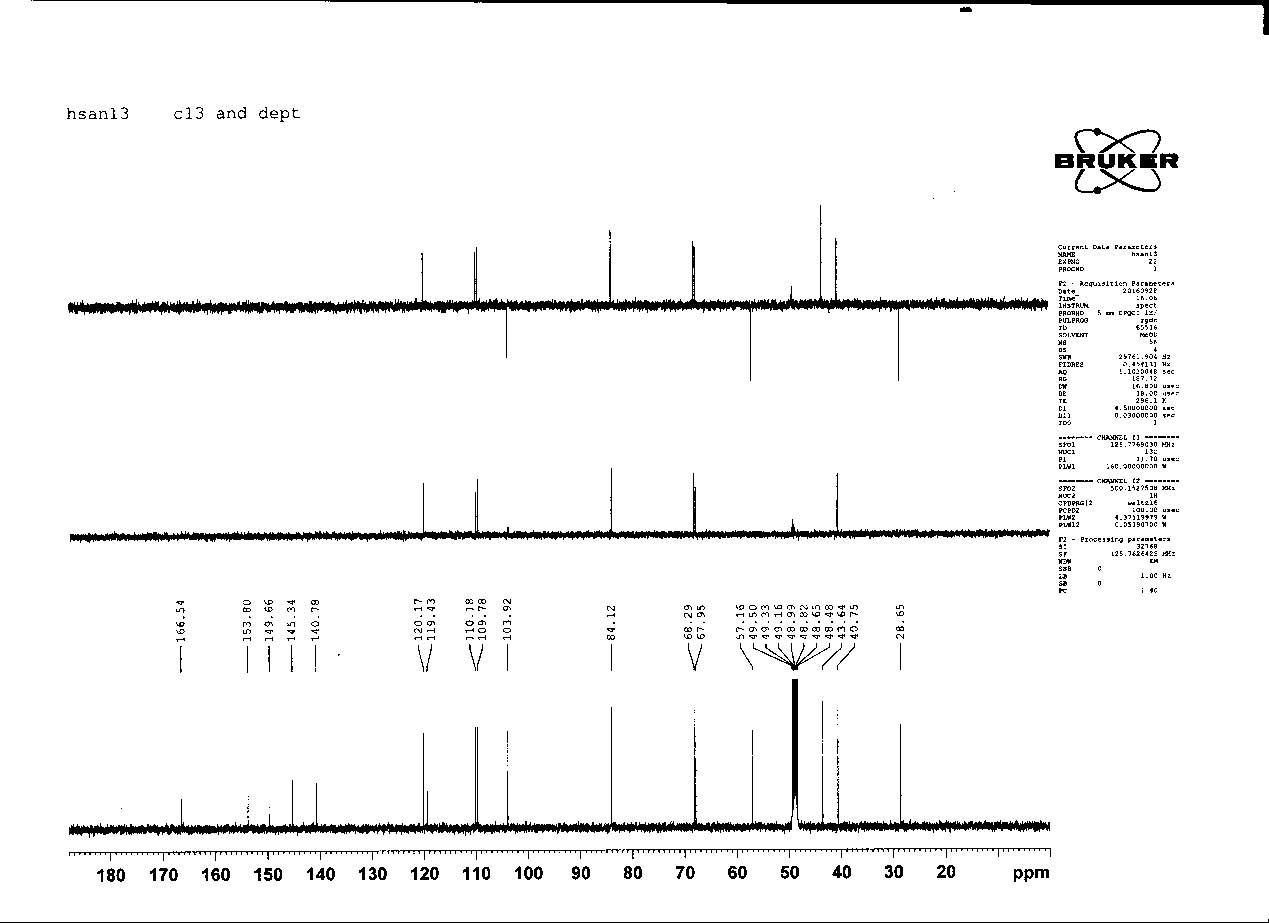


Figure S17. ^1^C NMR spectrum of Hippeastrine (6) in MeOD


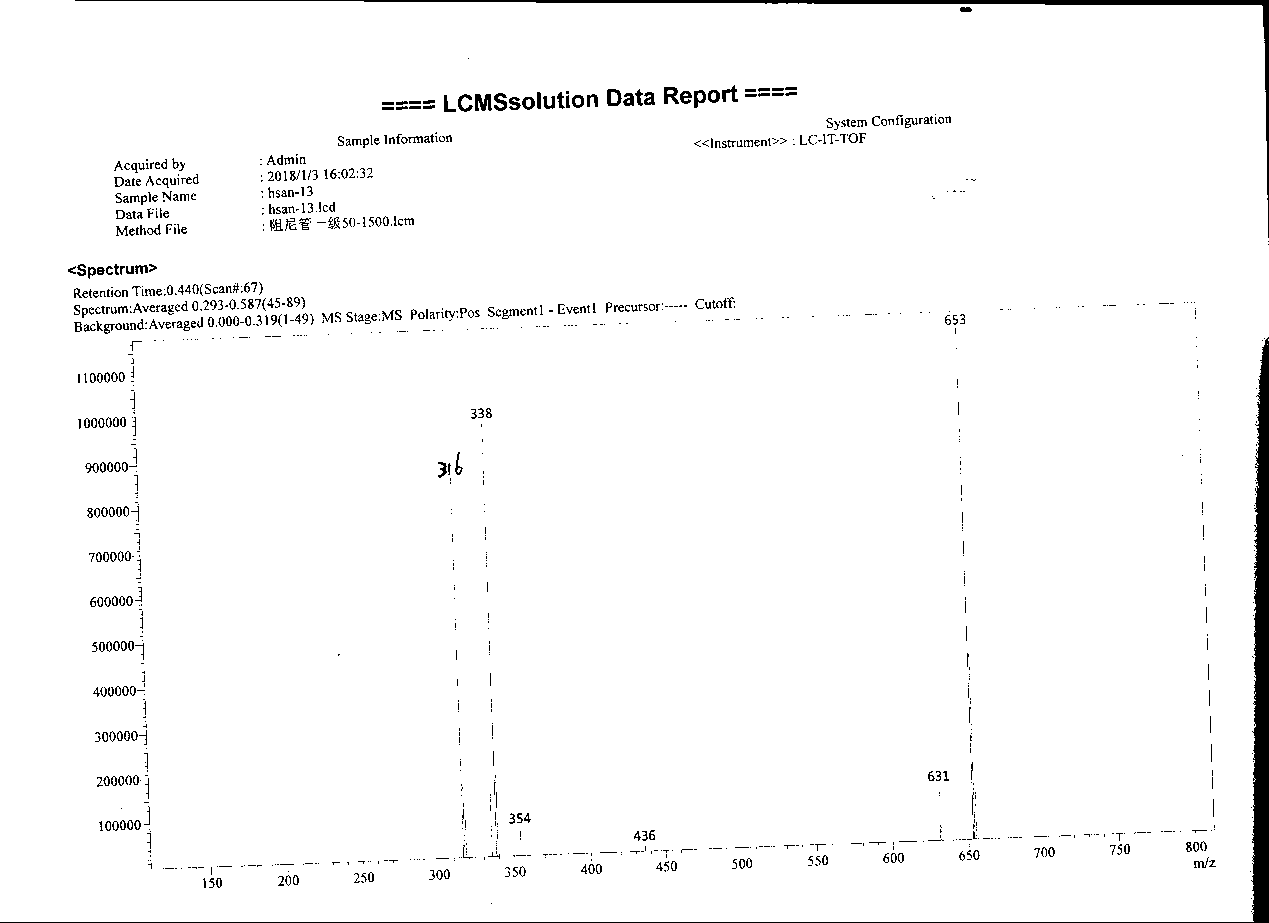


Figure S18. MS spectrum of Hippeastrine (6) in MeOD


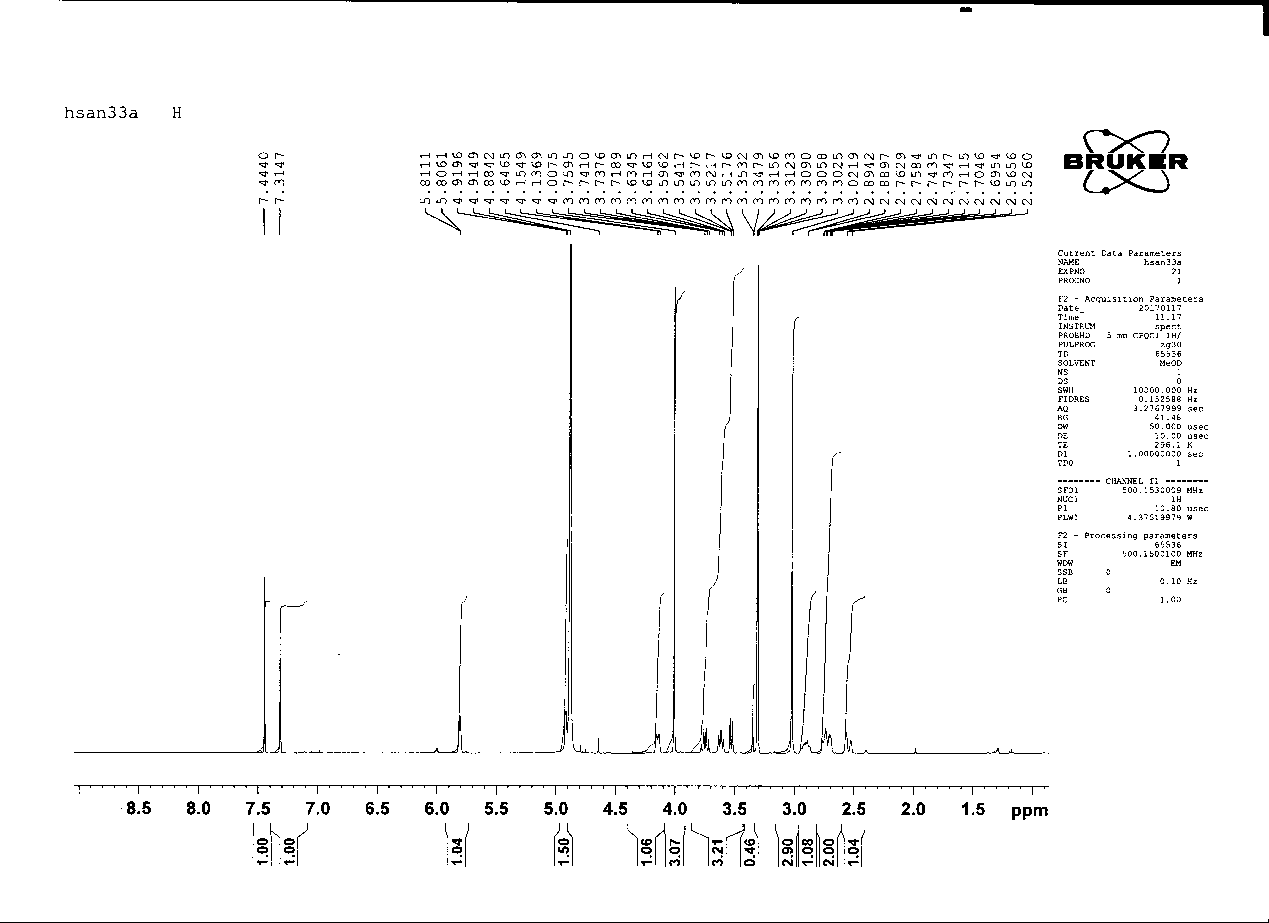


Figure S19. ^1^H NMR spectrum of O-Demethylhomolycorine N-oxide (7) in MeOD


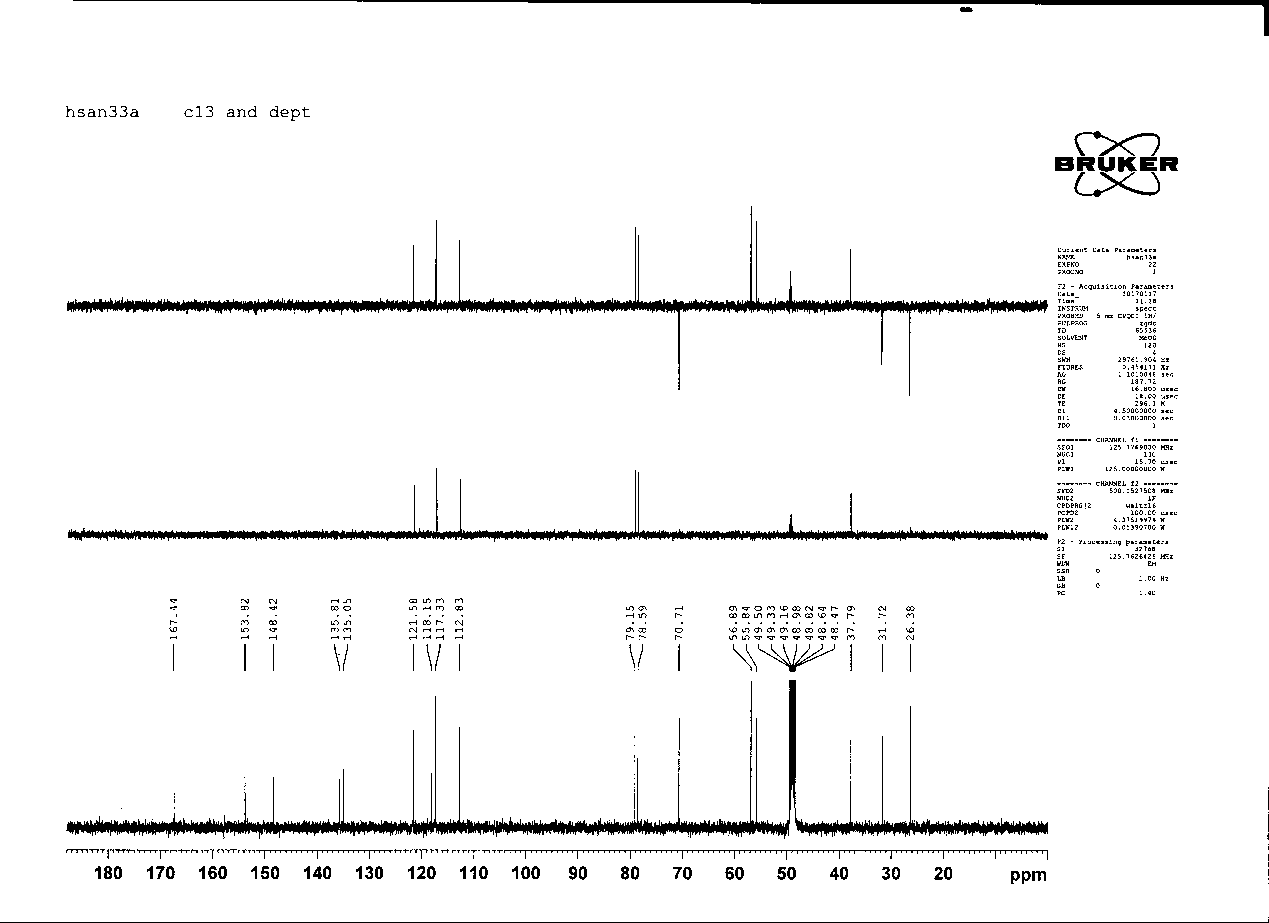


Figure S20. ^1^C NMR spectrum of O-Demethylhomolycorine N-oxide (7) in MeOD


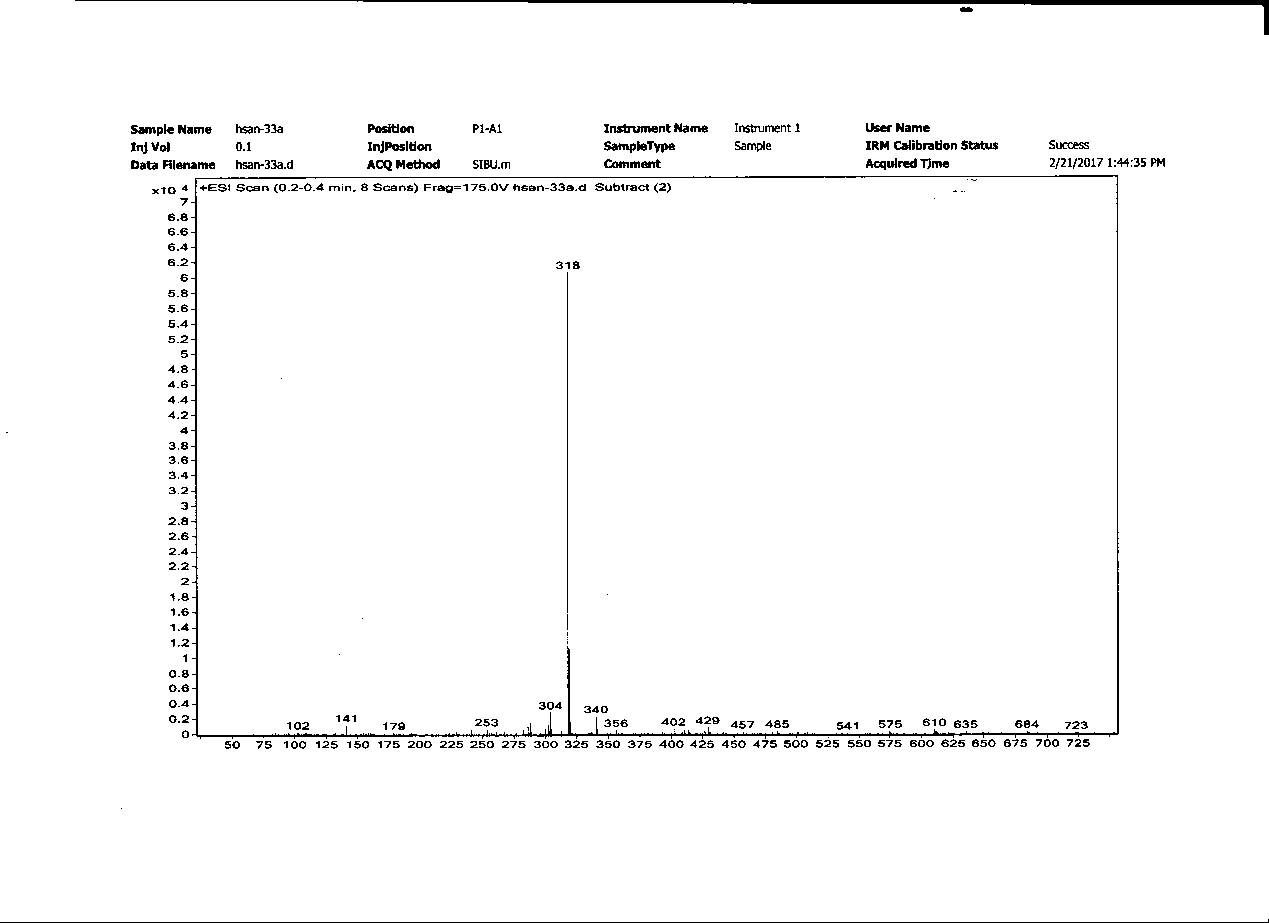


Figure S21. MS spectrum of O-Demethylhomolycorine N-oxide (7) in MeOD


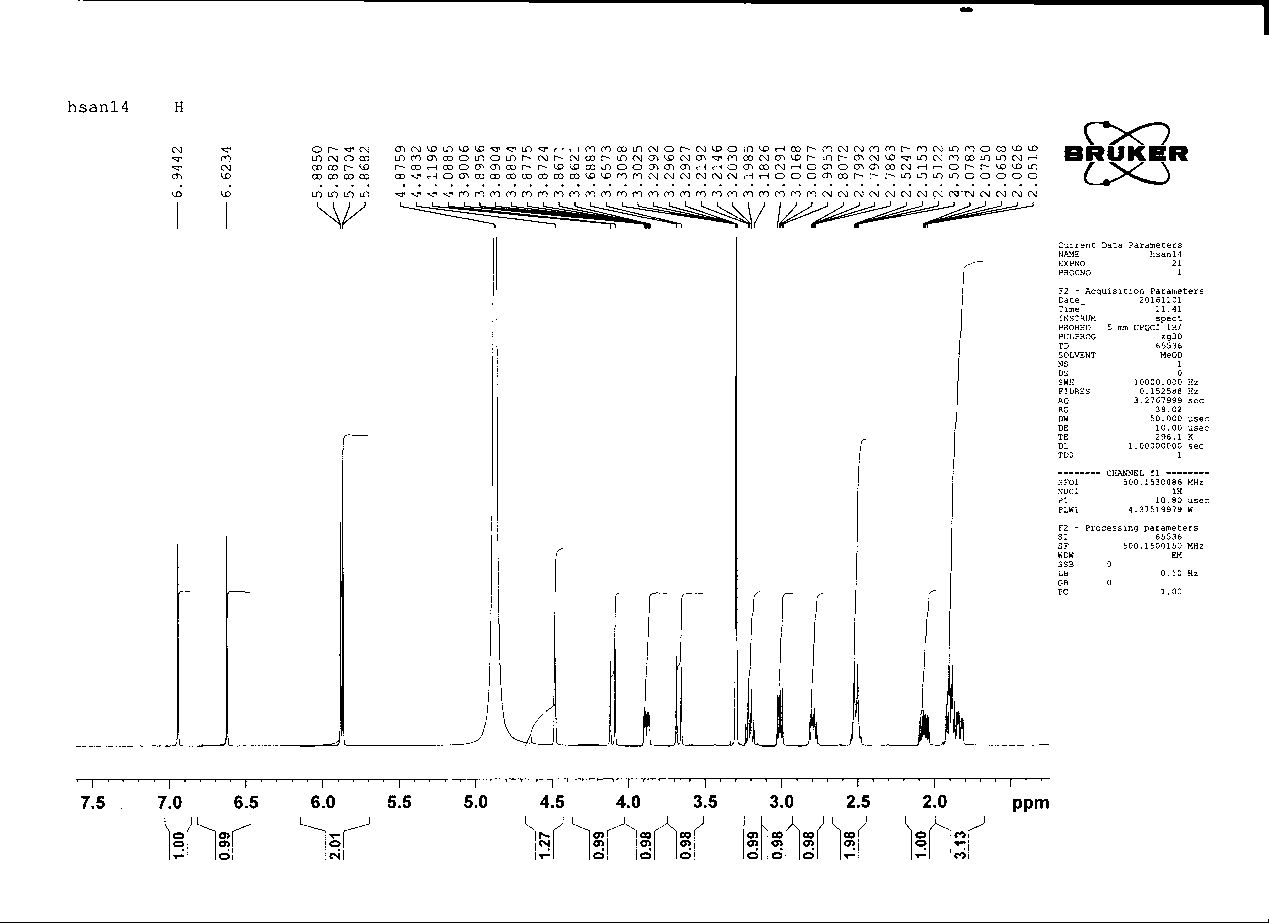


Figure S22. ^1^H NMR spectrum of Zephyranthine (8) in MeOD


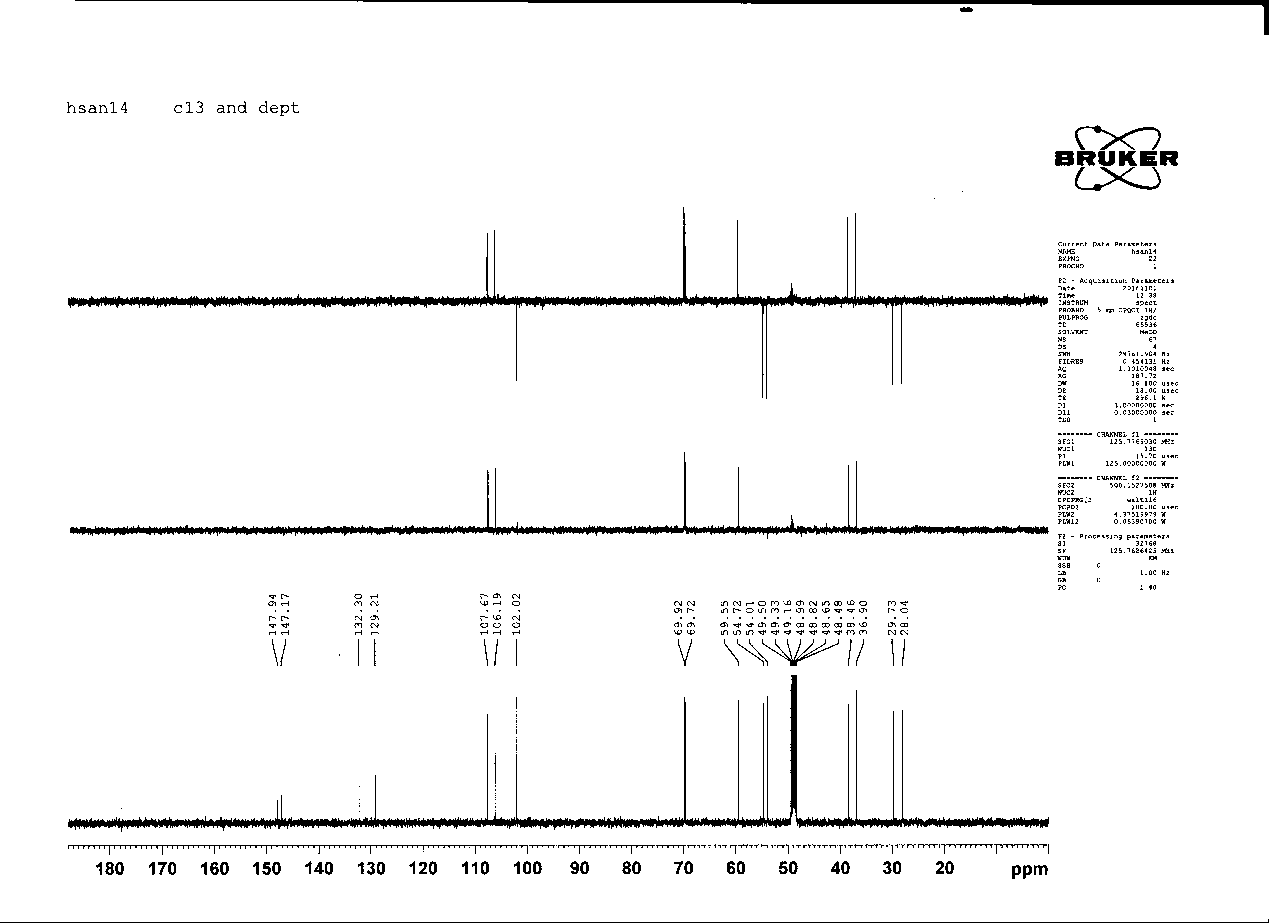


Figure S23. ^1^C NMR spectrum of Zephyranthine (8) in MeOD


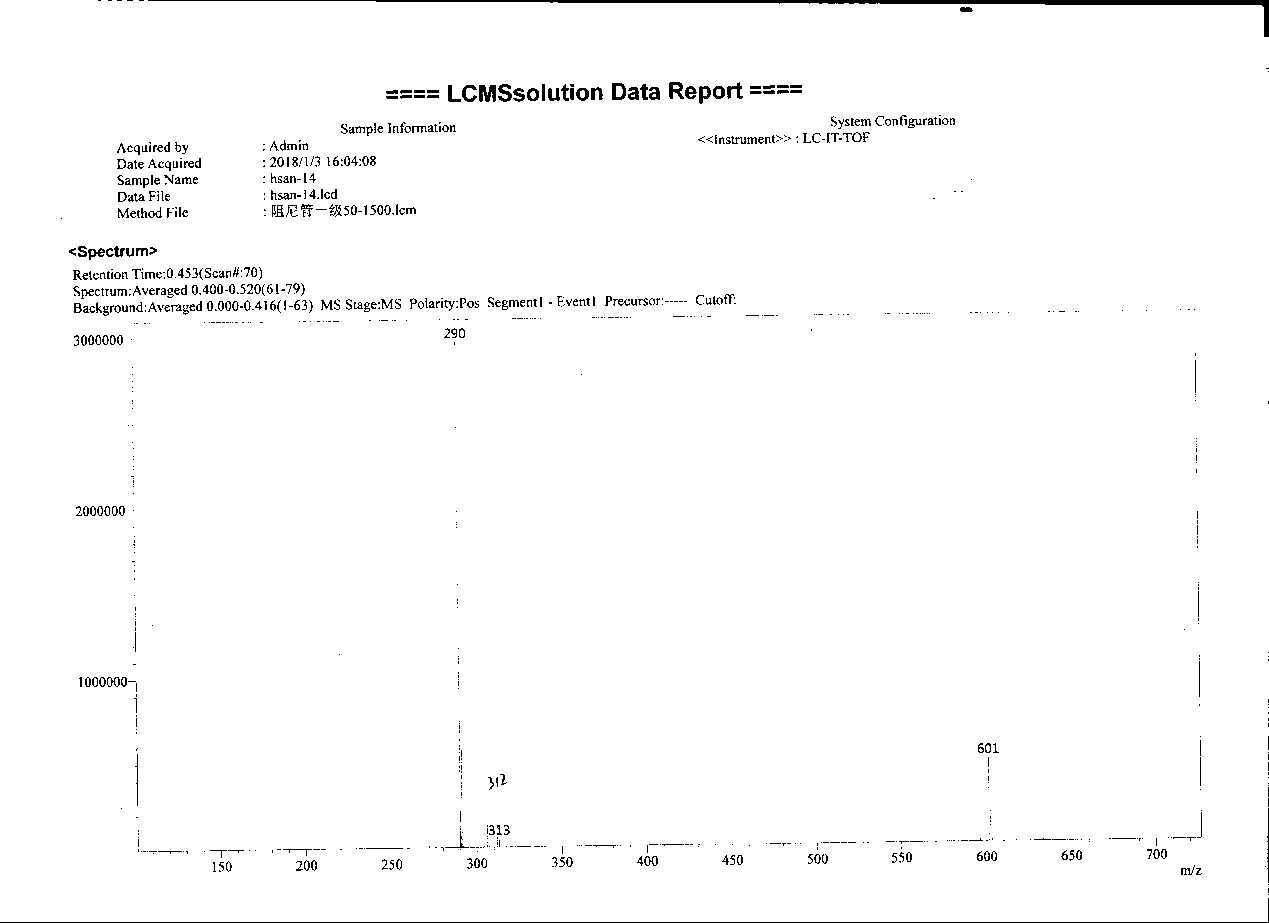


Figure S24. MS spectrum of Zephyranthine (8) in MeOD


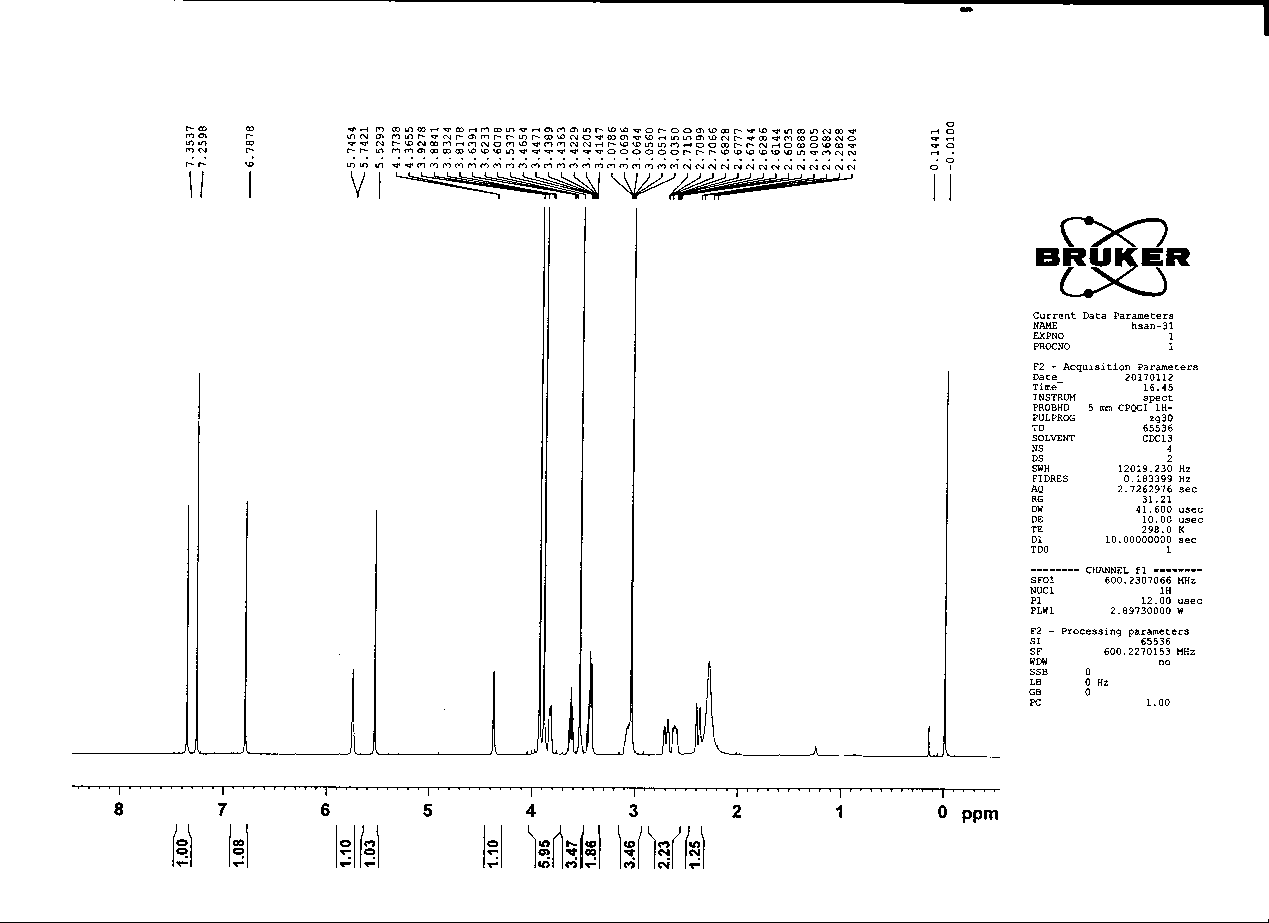


Figure S25. ^1^H NMR spectrum of O-Methyllycorenine N-oxide (9) in CDCl_3_


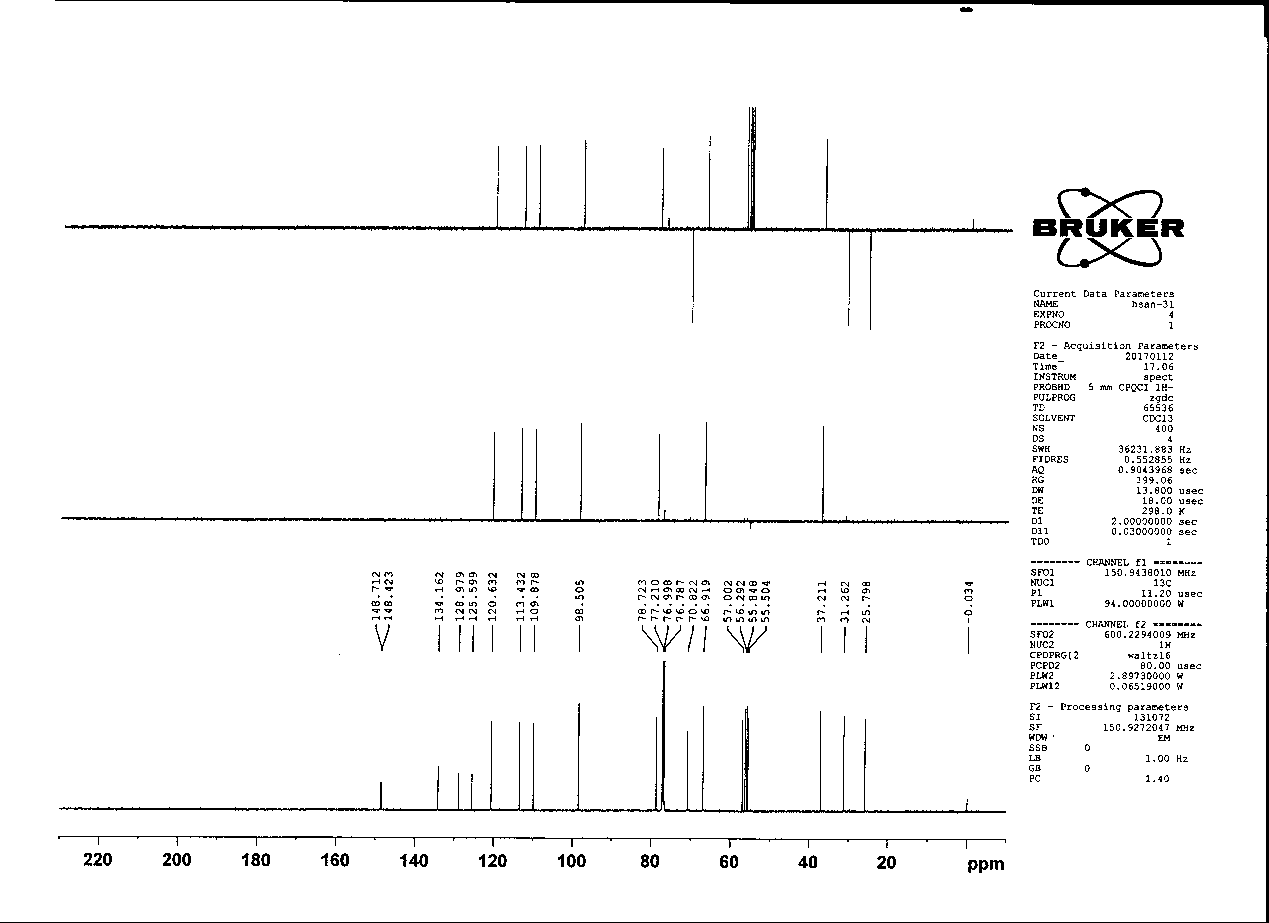


Figure S26. ^1^C NMR spectrum of O-Methyllycorenine N-oxide (9) in CDCl_3_


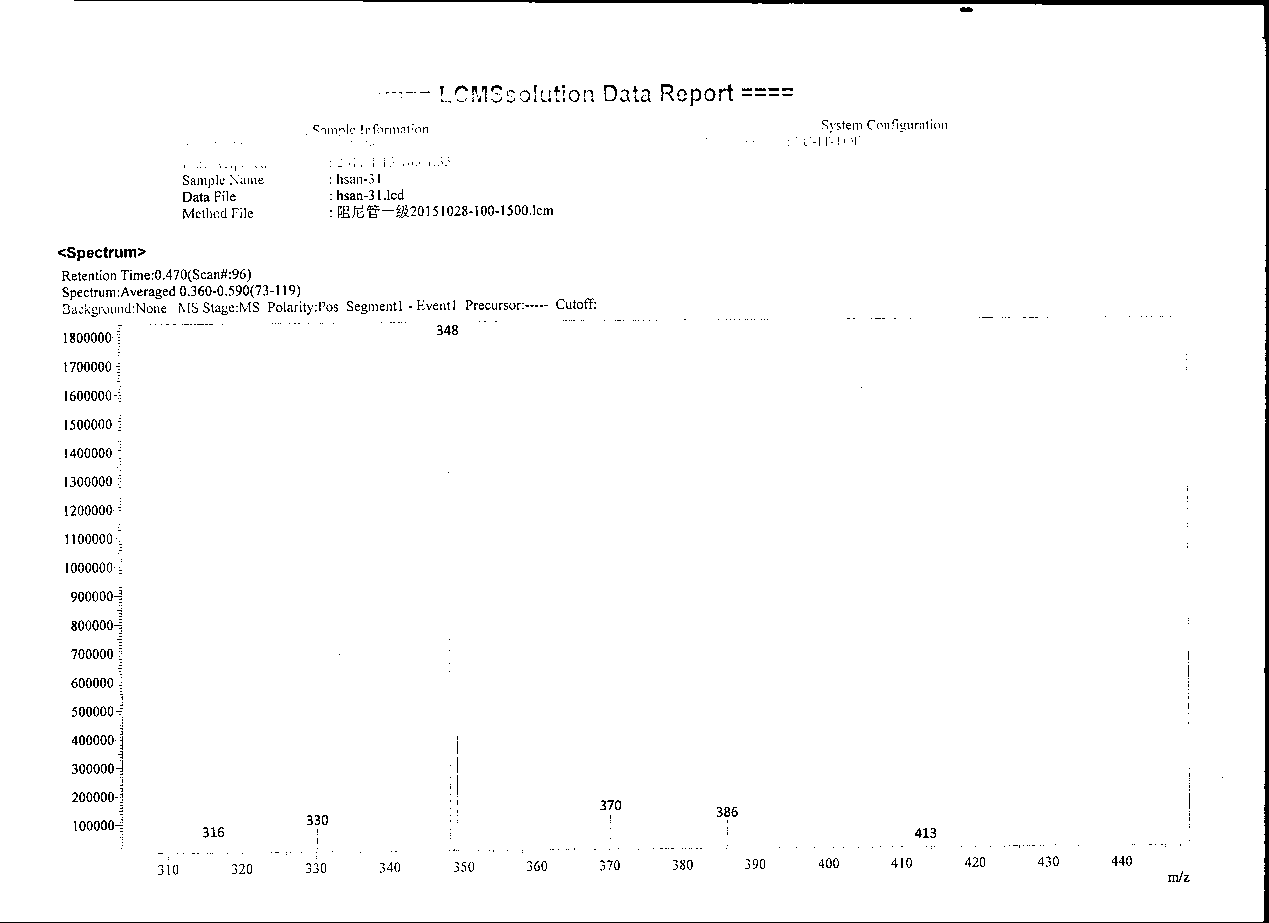


Figure S27. MS spectrum of O-Methyllycorenine N-oxide (9) in CDCl_3_


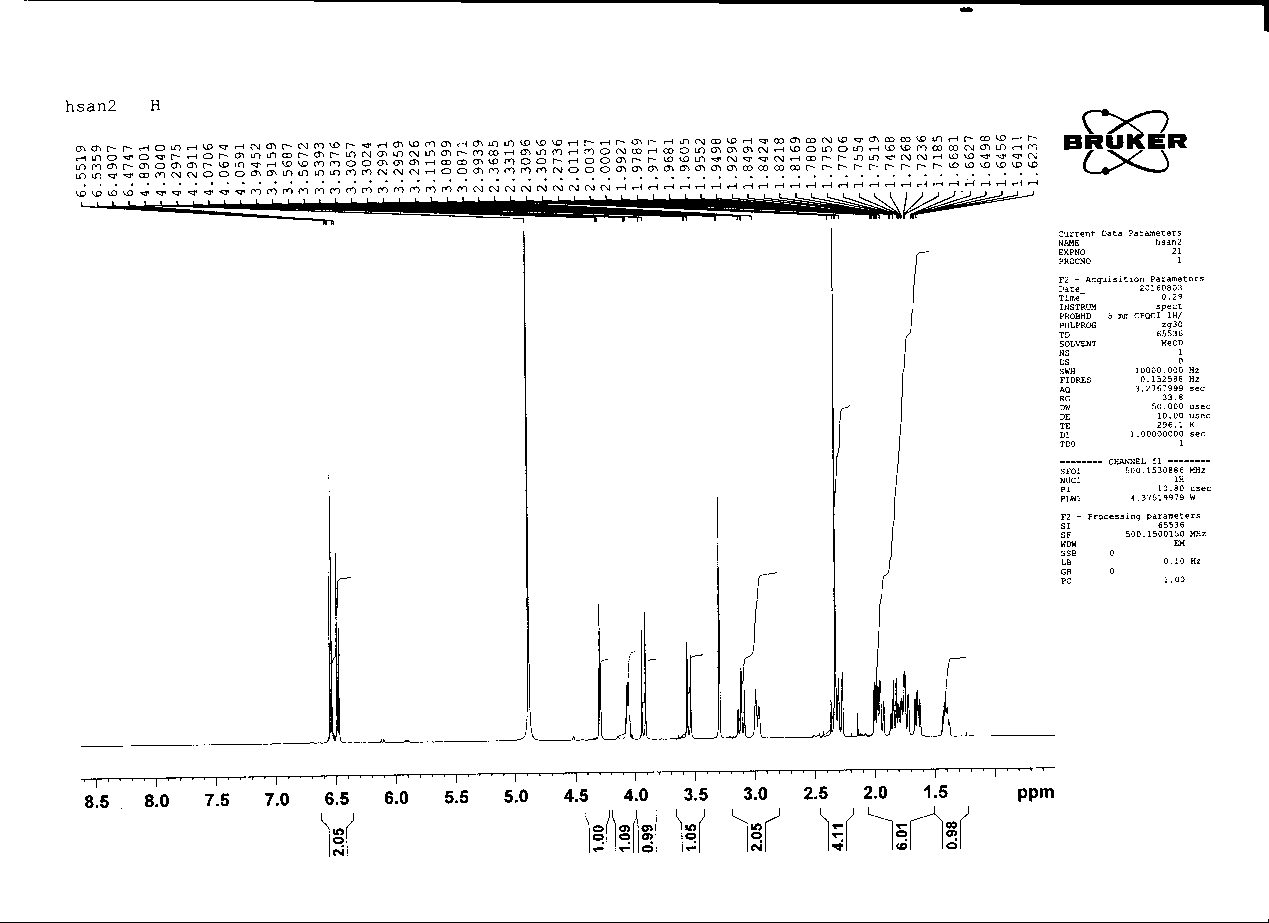


Figure S28. ^1^H NMR spectrum of Lycoranine C (10) in MeOD


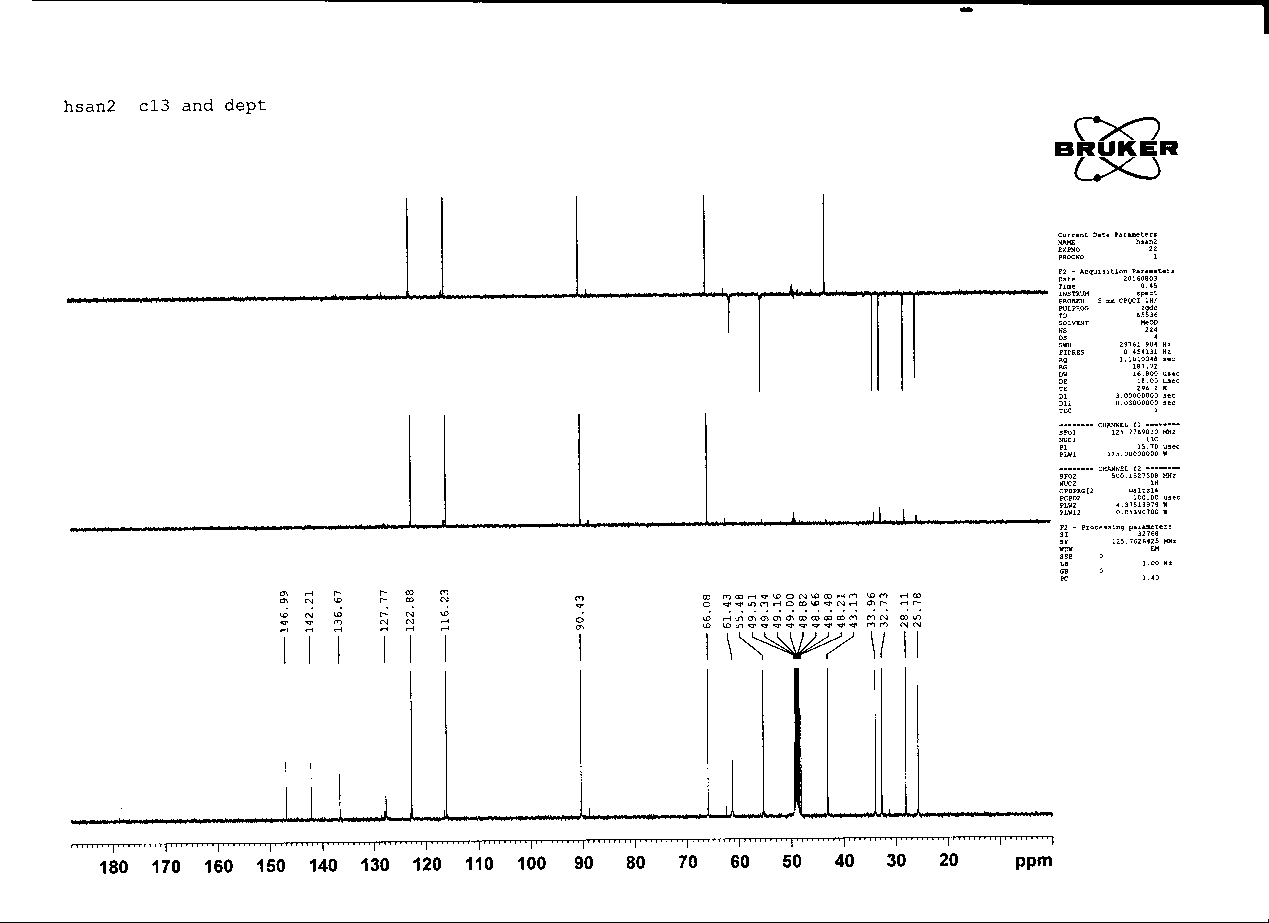


Figure S29. ^1^C NMR spectrum of Lycoranine C (10) in MeOD


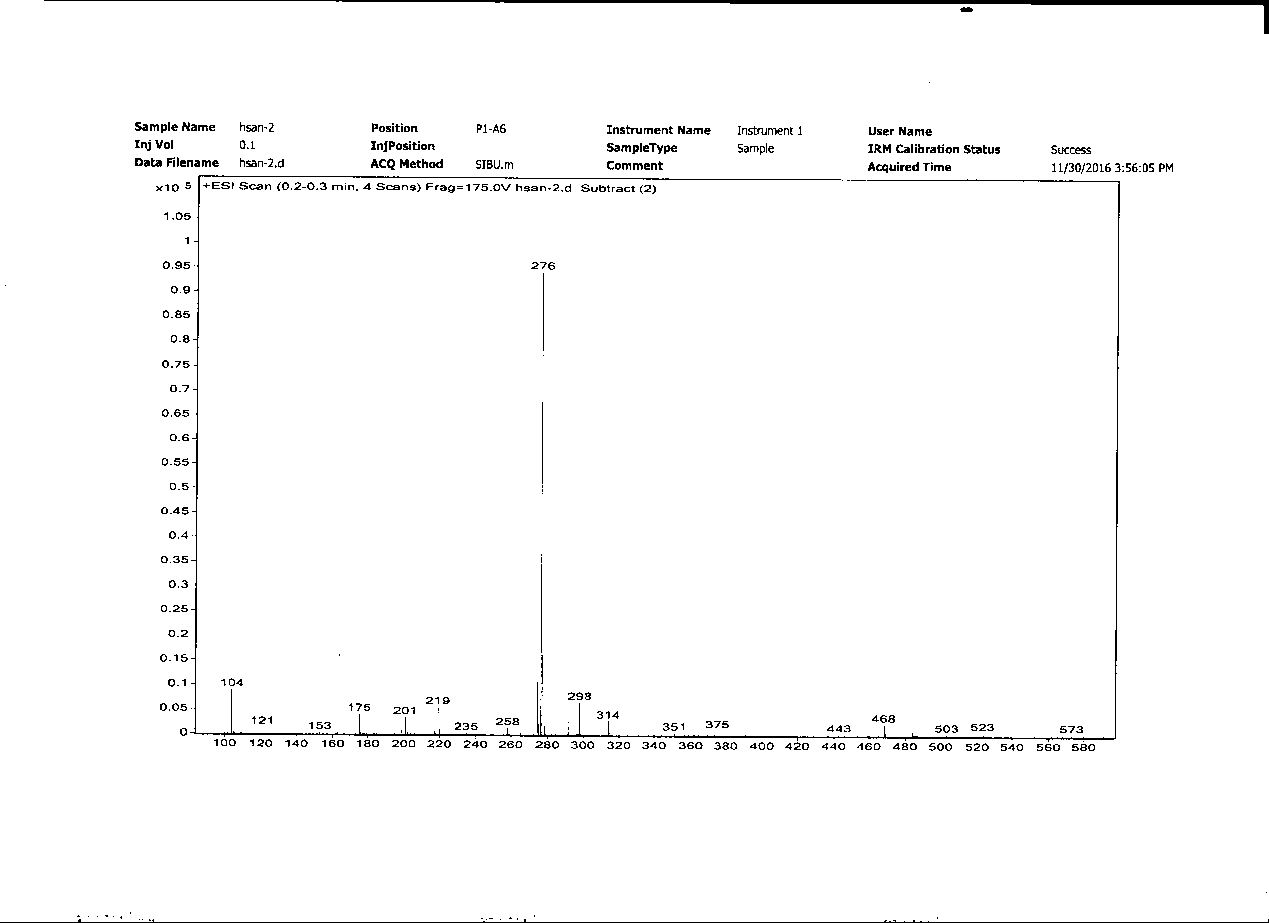


Figure S30. MS spectrum of Lycoranine C (10) in MeOD


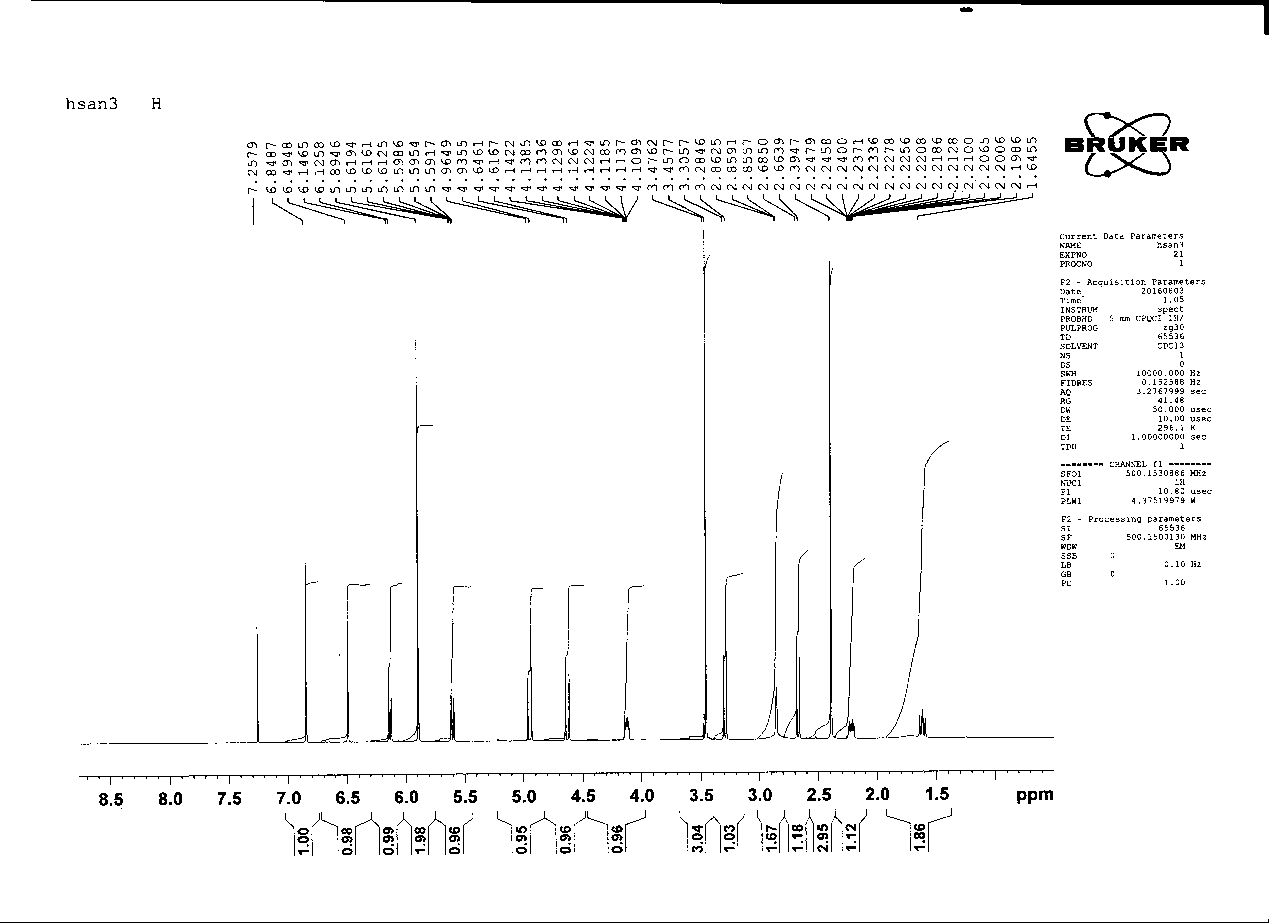


Figure S31. ^1^H NMR spectrum of Tazettine (11) in CDCl_3_


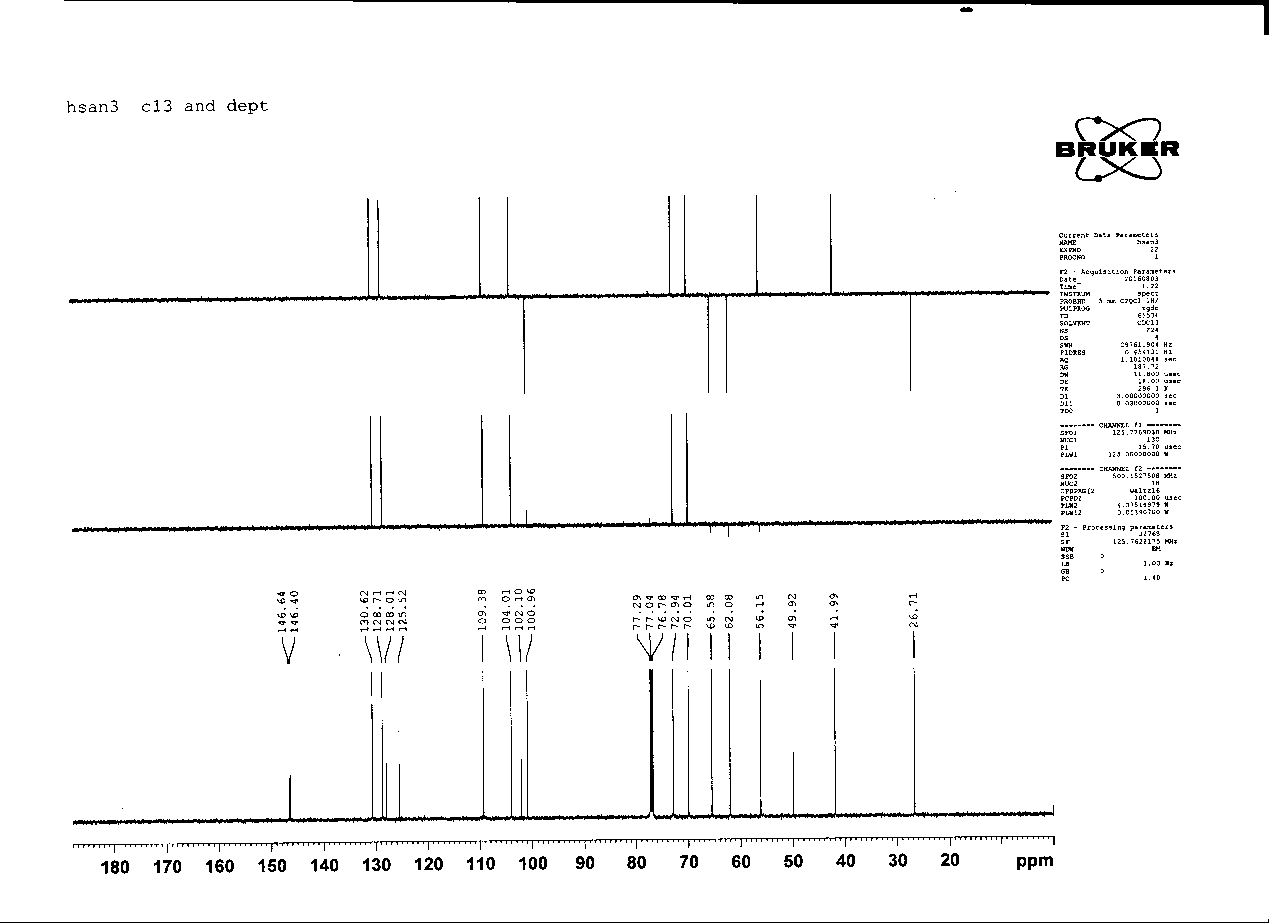


Figure S32. ^1^C NMR spectrum of Tazettine (11) in CDCl_3_


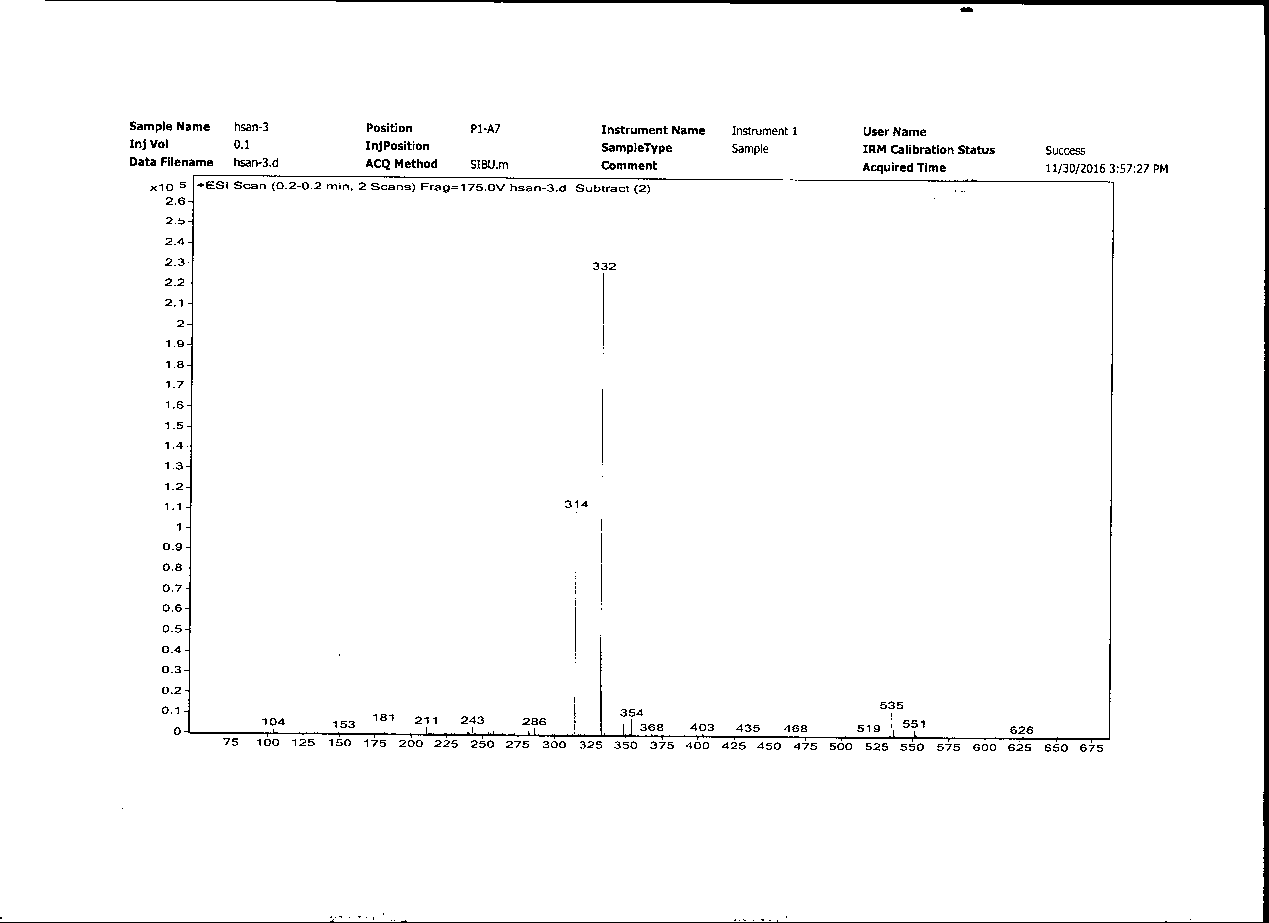


Figure S33. MS spectrum of Tazettine (11) in CDCl_3_


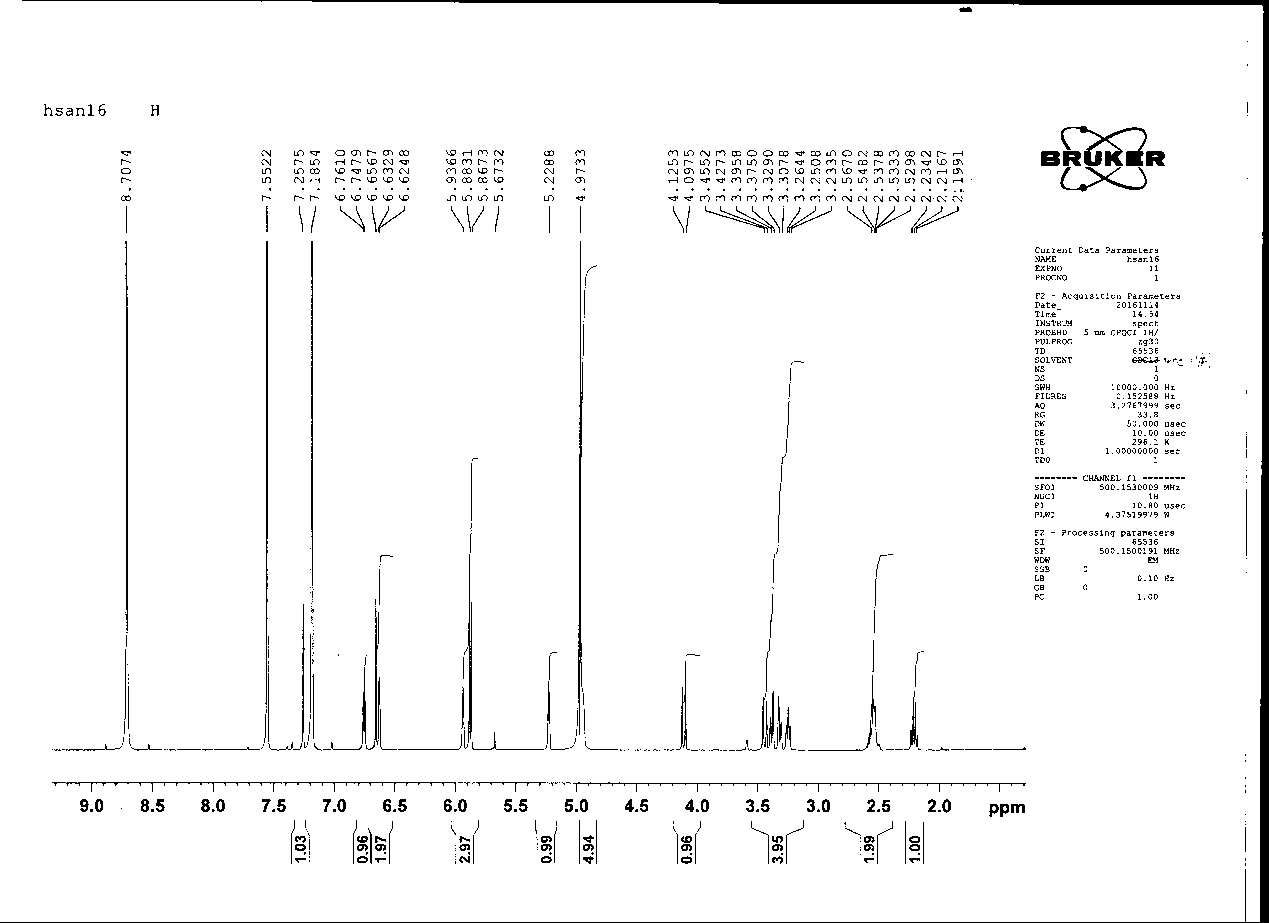


Figure S34. ^1^H NMR spectrum of Lycorine (12) in CDCl_3_


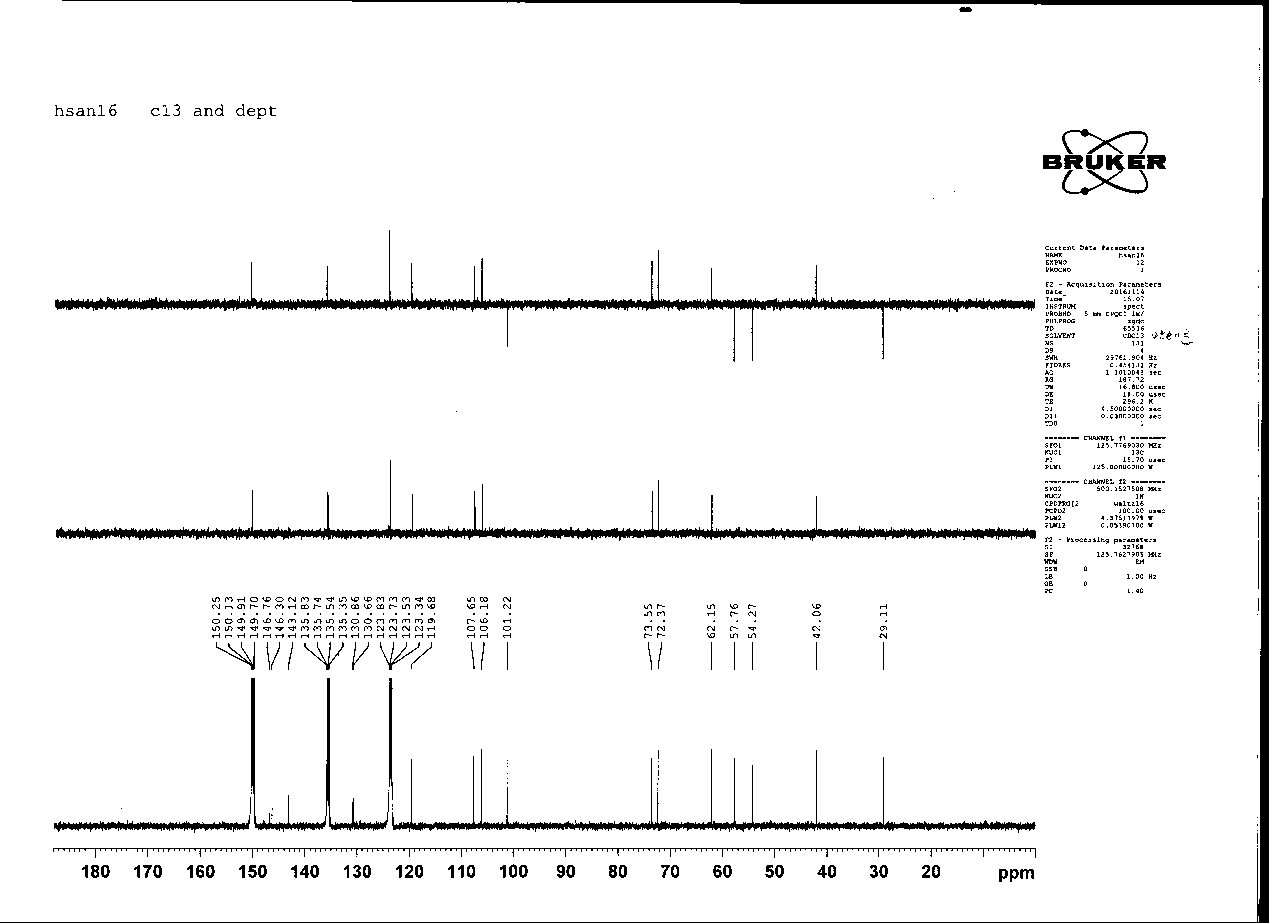


Figure S35. ^1^C NMR spectrum of Lycorine (12) in CDCl_3_


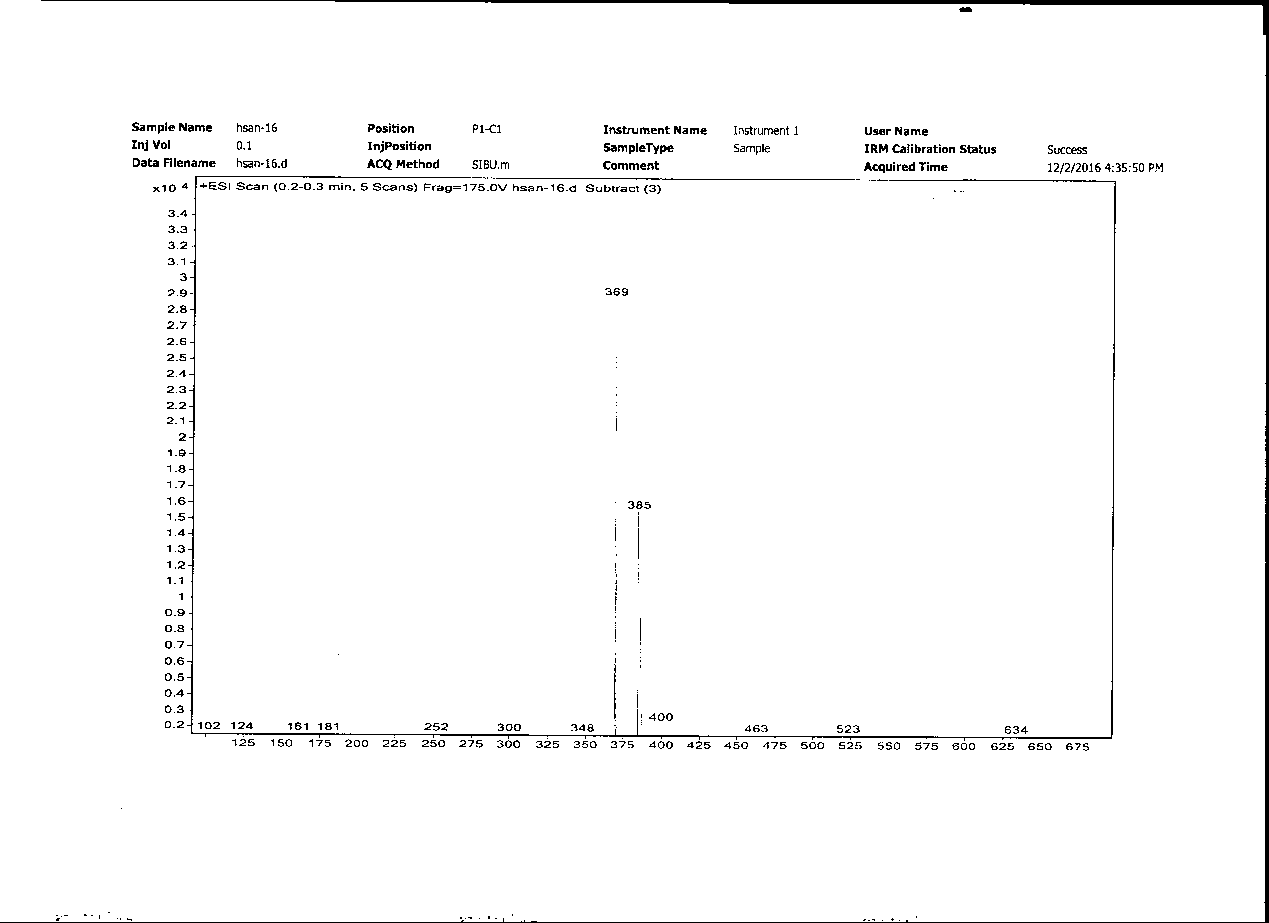


Figure S36. MS spectrum of Lycorine (12) in CDCl_3_


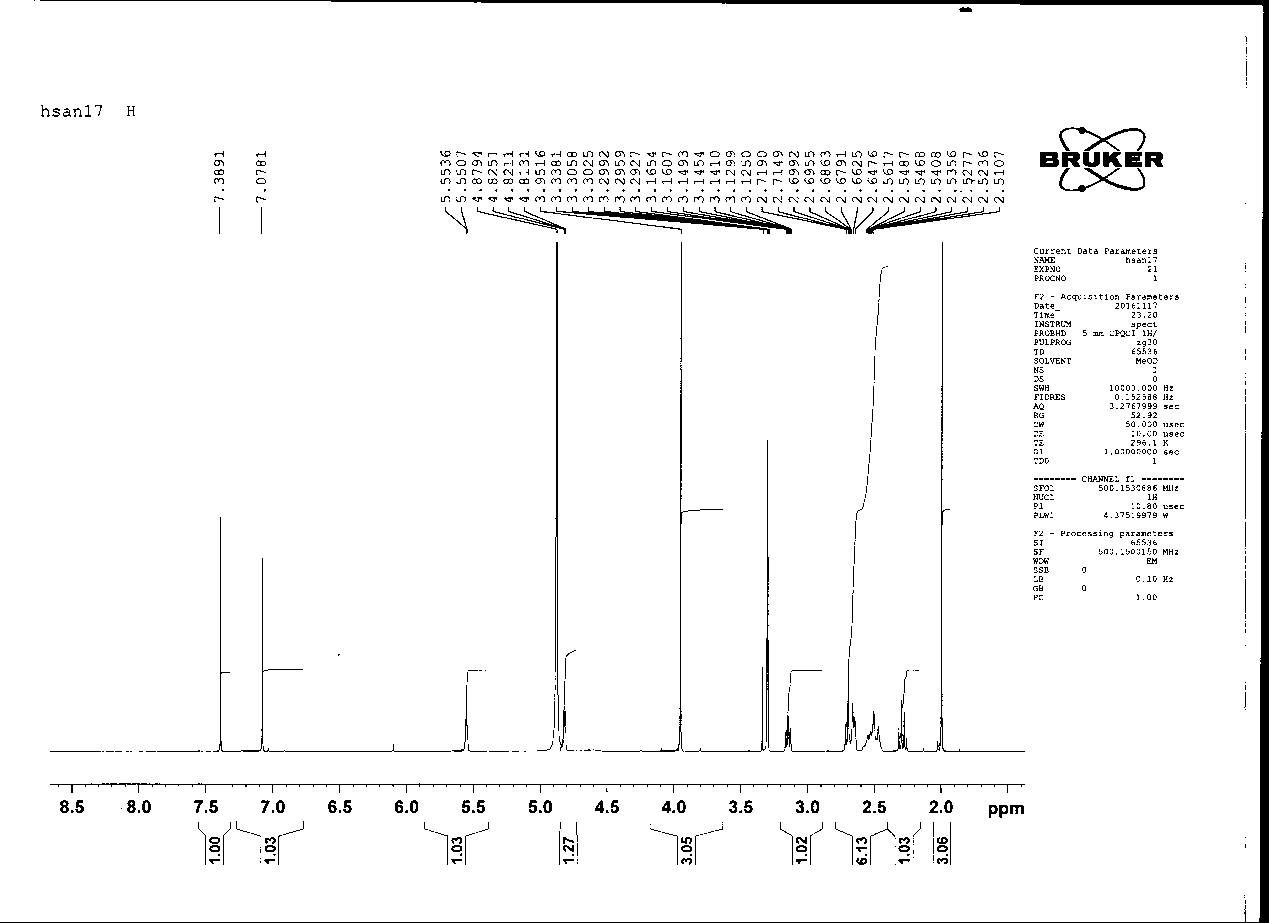


Figure S37. ^1^H NMR spectrum of 9-O-Demethylhomolycorine (13) in MeOD


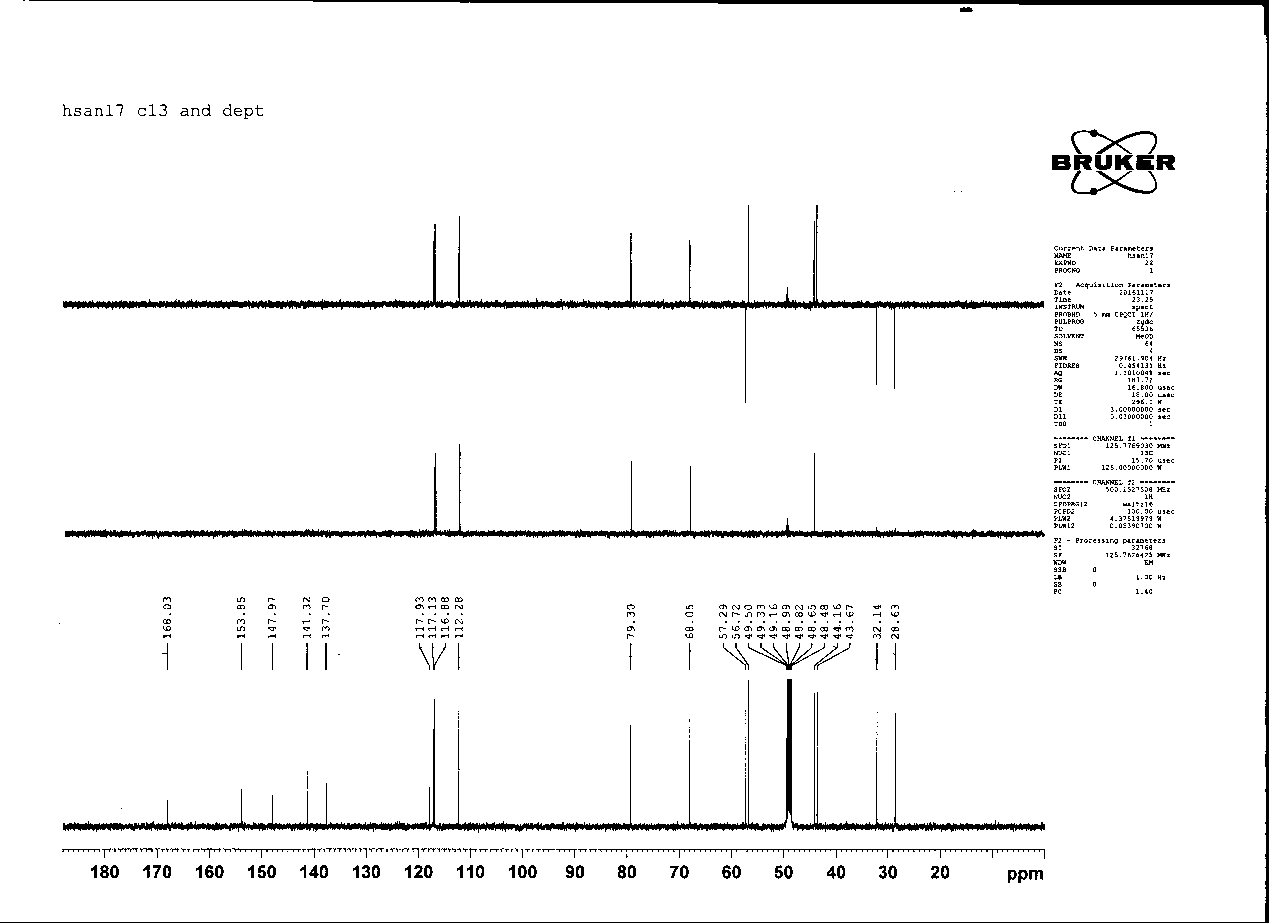


Figure S38. ^1^C NMR spectrum of 9-O-Demethylhomolycorine (13) in MeOD


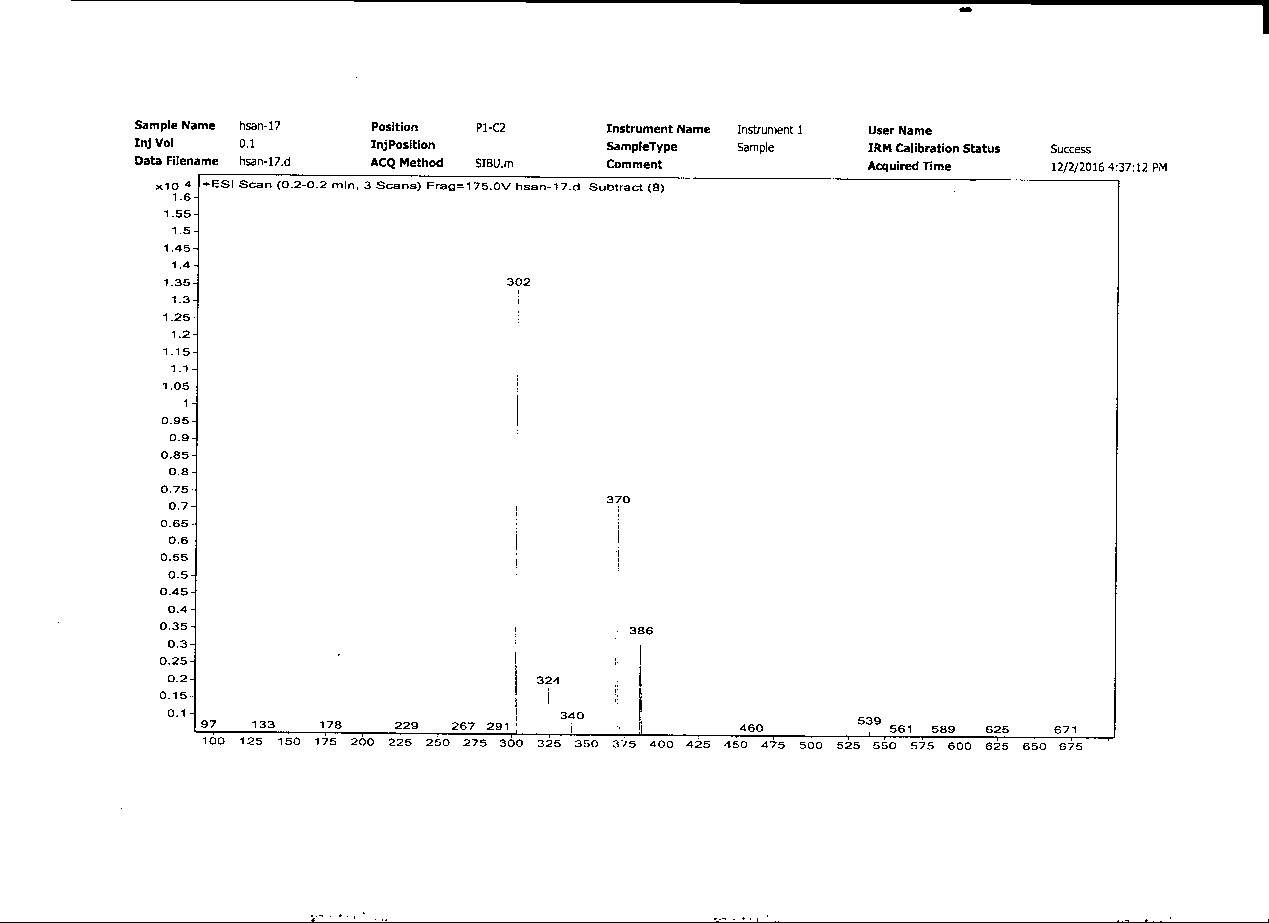


Figure S39. MS spectrum of 9-O-Demethylhomolycorine (13) in MeOD


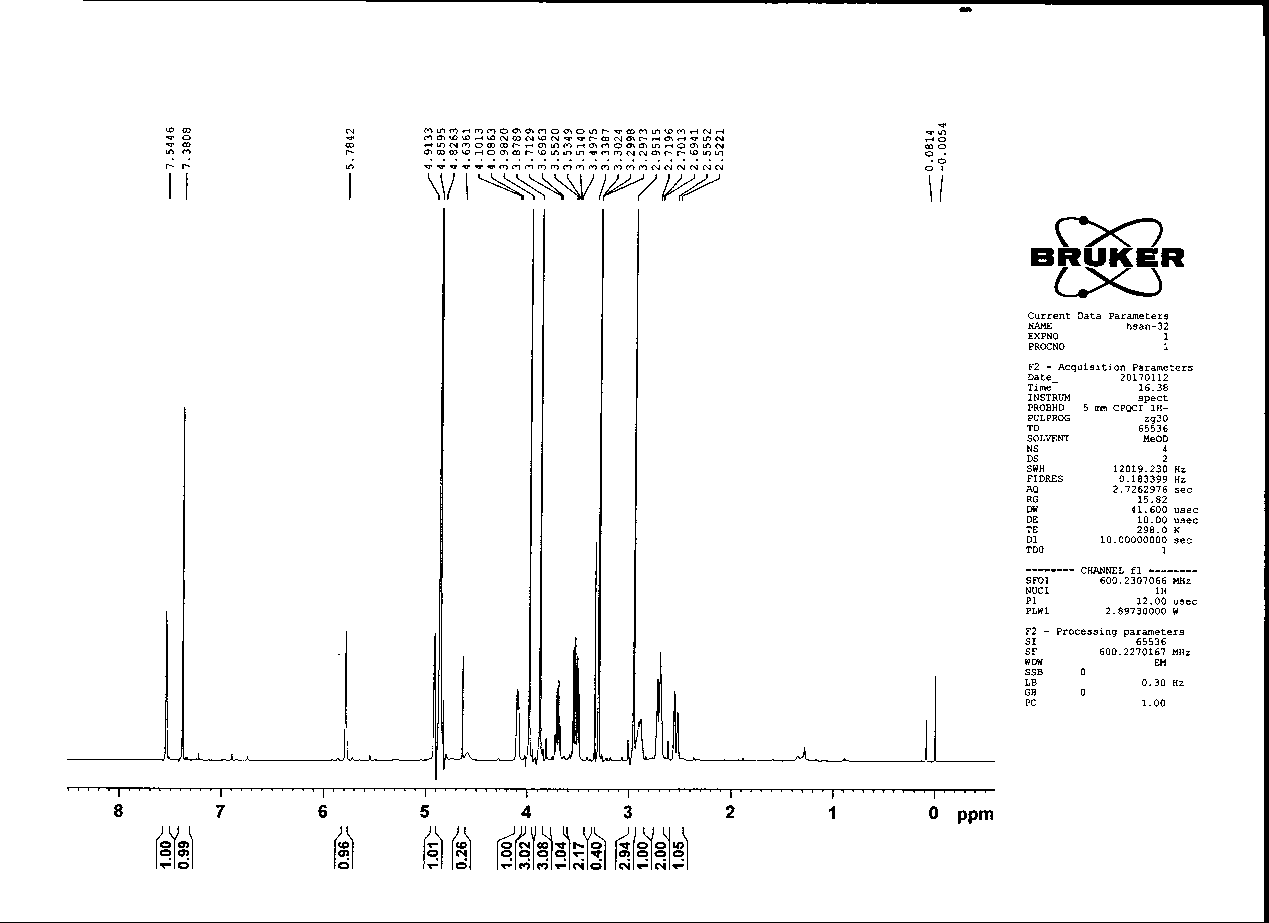


Figure S40. ^1^H NMR spectrum of Homolycorine N-oxide (14) in MeOD


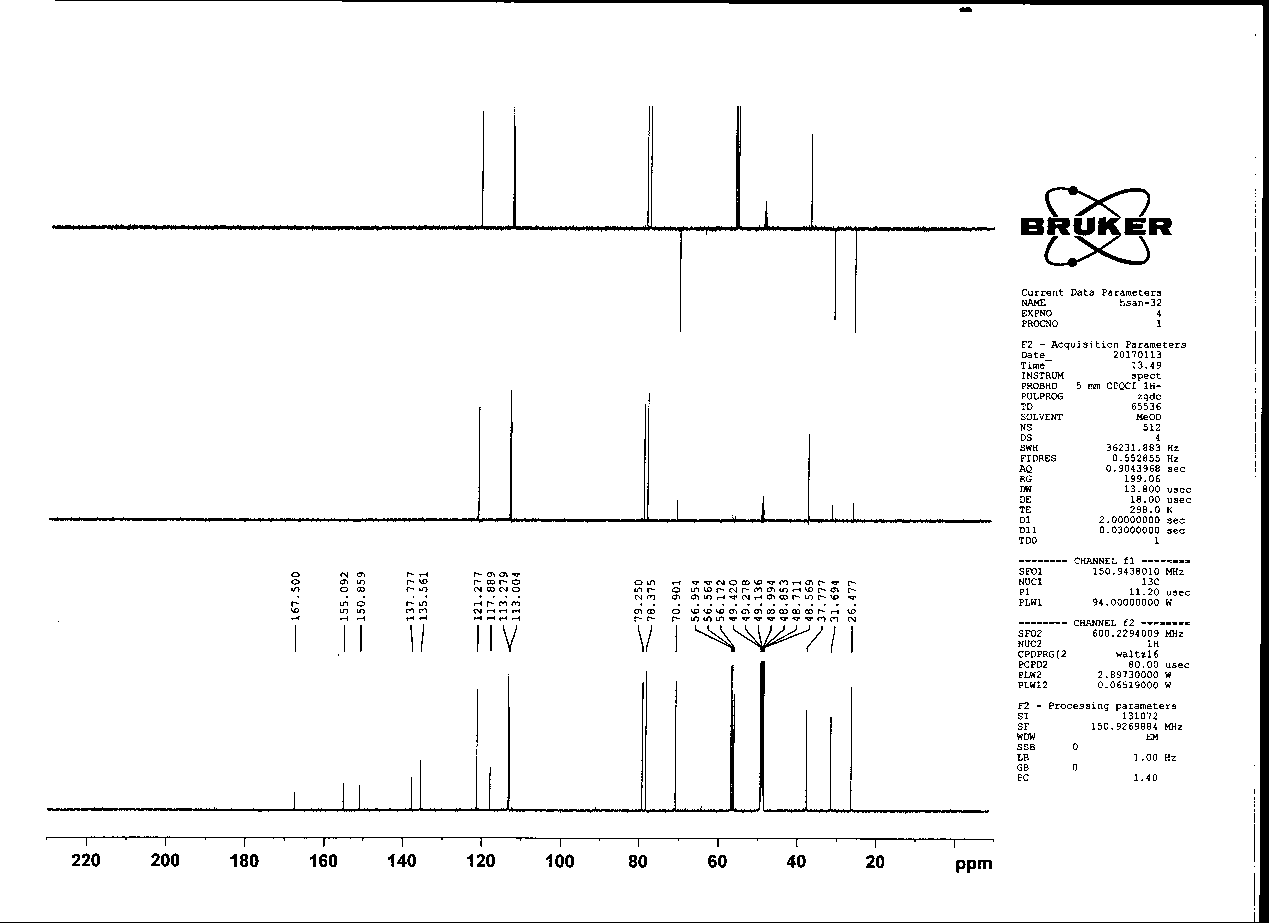


Figure S41. ^1^C NMR spectrum of Homolycorine N-oxide (14) in MeOD


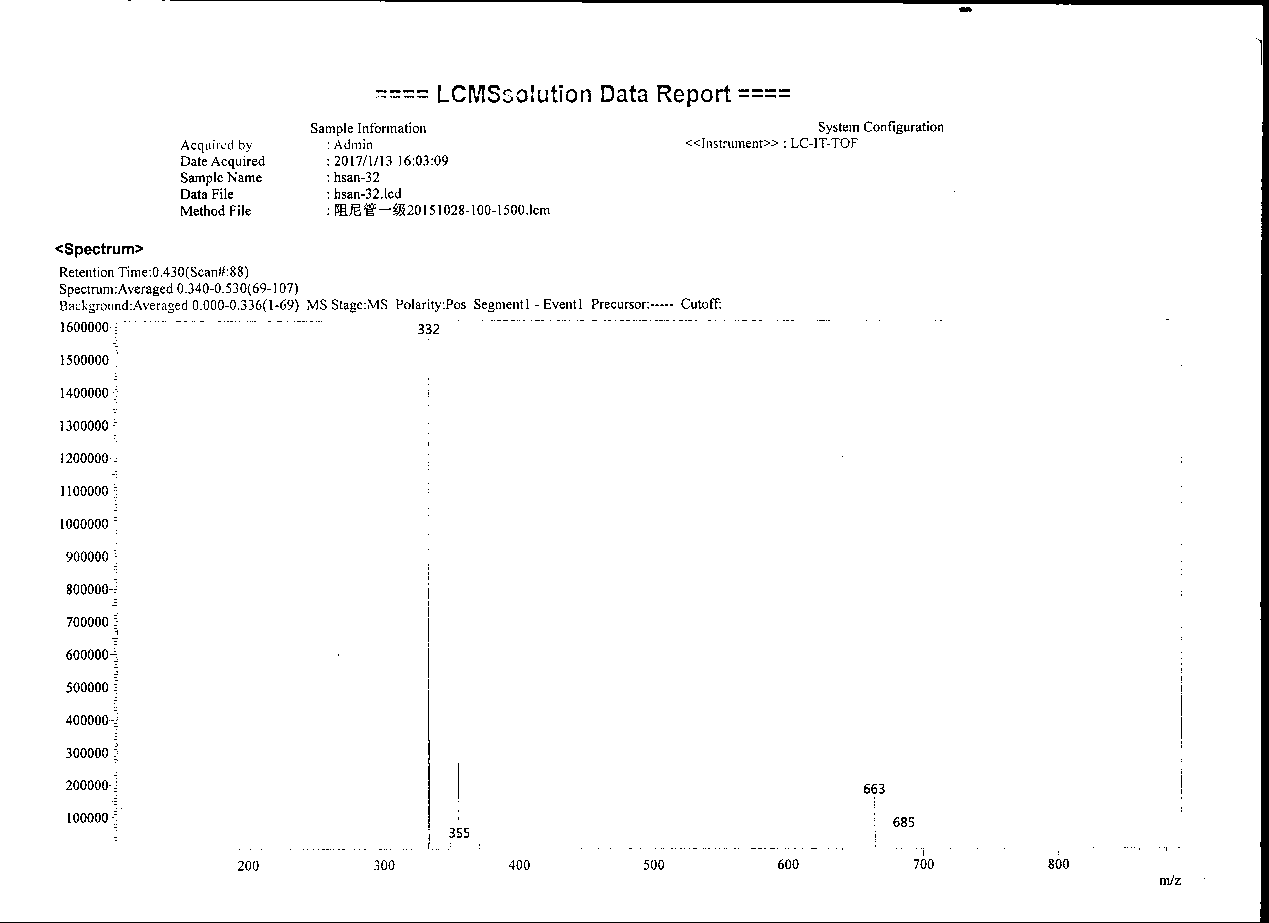


Figure S42. MS spectrum of Homolycorine N-oxide (14) in MeOD


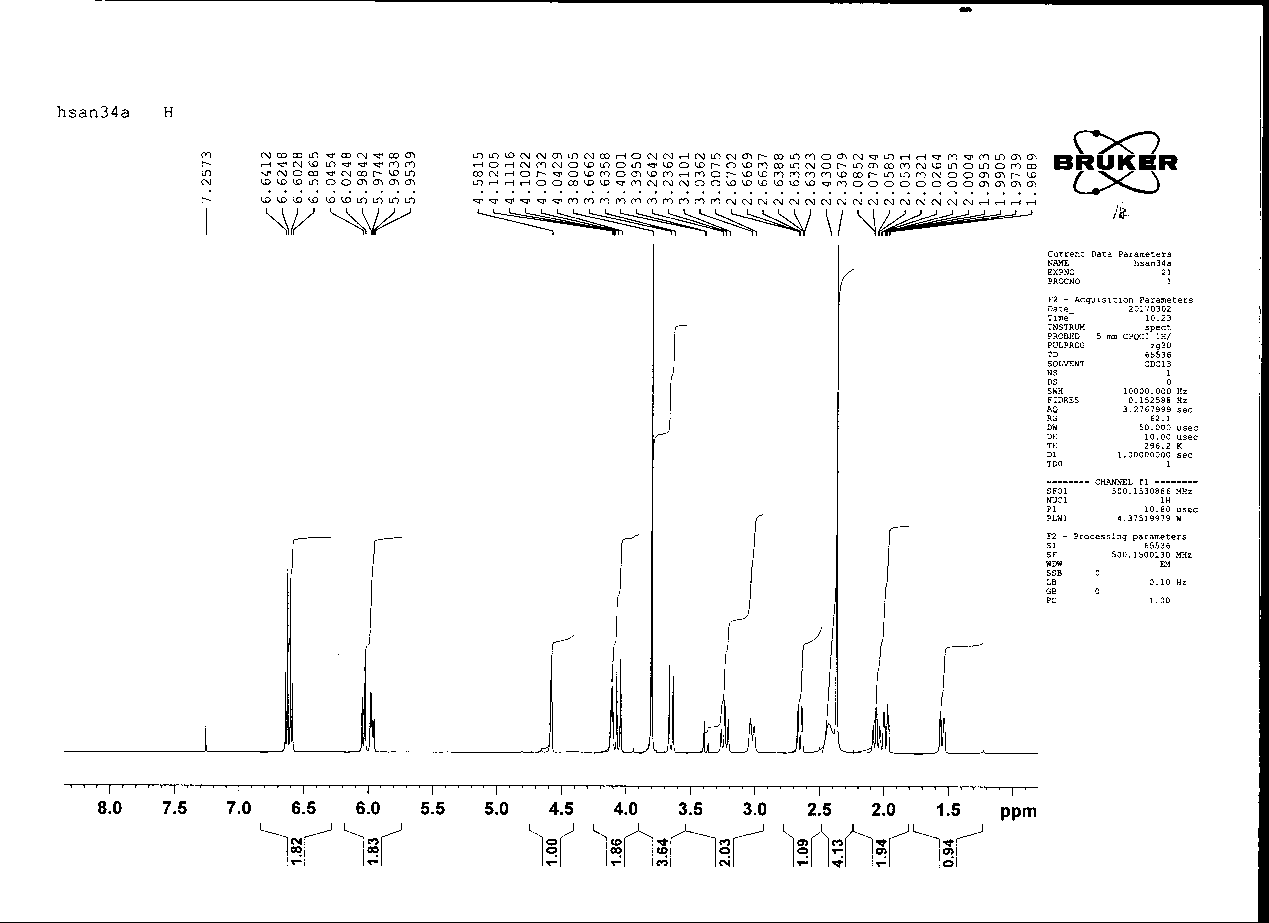


Figure S43. ^1^H NMR spectrum of Galanthamine (15) in CDCl_3_


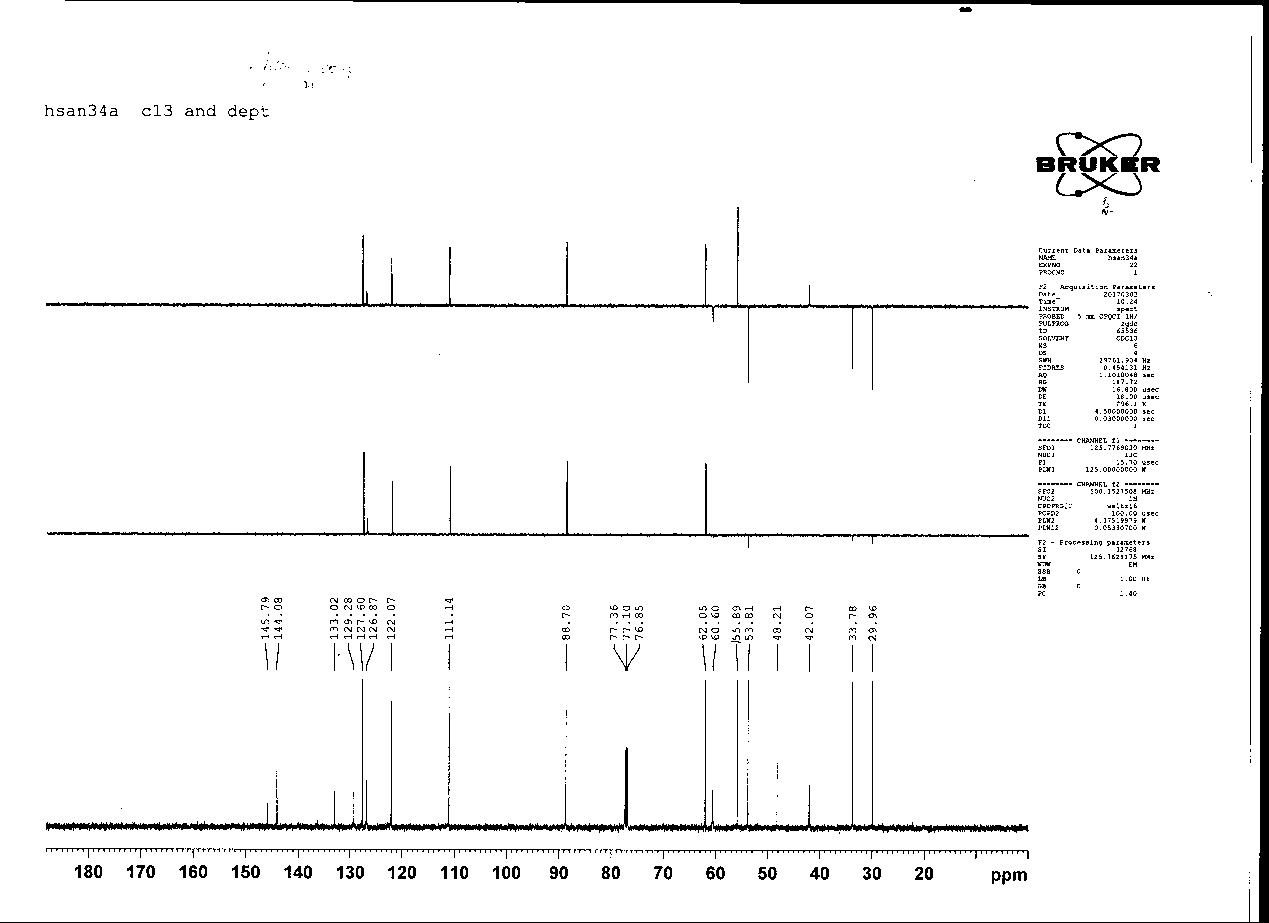


Figure S44. ^1^C NMR spectrum of Galanthamine (15) in CDCl_3_


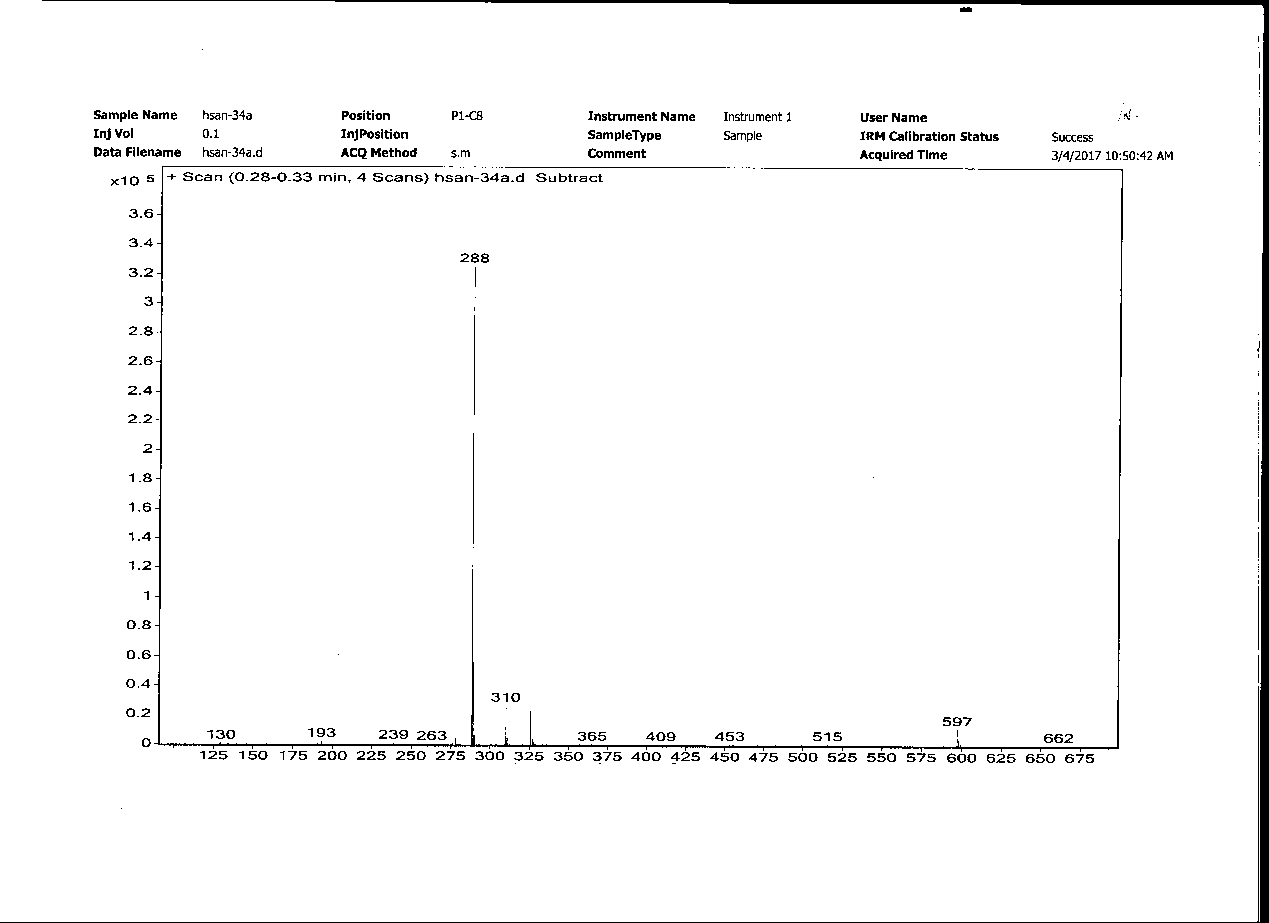


Figure S45. MS spectrum of Galanthamine (15) in CDCl_3_
